# An extended approach to value chain analysis

**DOI:** 10.1186/s40008-021-00244-6

**Published:** 2021-08-02

**Authors:** Klemen Knez, Andreja Jaklič, Metka Stare

**Affiliations:** Centre of International Relations, University of Ljubljana, Ljubljana, Slovenia

**Keywords:** Value chain typology, Value chains, Global value chain, Domestic value chain, Participation rate, Input–output framework, F1, F4, F6

## Abstract

In the article, we propose a comprehensive methodology of value chain analysis in the international input–output framework that introduces a new measure of value chain participation and an extended typology of value chains, with the novel inclusion of domestic value chain to address the extent of fragmentation of purely domestic production. This allows for the simultaneous analysis of both global and domestic production fragmentation, the complex patterns of their evolution and their impact on economic development. The main contribution of the proposed methodology is conceptual: it permits the measurement of all value chain paths that pass through each country-sector from production to final consumption, whether the path includes downstream linkages, upstream linkages or their combination. Empirical application of this methodology shows the importance of including domestic fragmentation in value chain analysis: The fragmentation of both global and domestic levels of production has a significant positive correlation with economic growth. This implies that the effects of global production fragmentation must be analysed together with the changing structure of the fragmentation of domestic production to obtain the whole picture, one that might provide important information for policymaking and industrial policy.

## Introduction

In recent decades, the growing complexity of the division of labour has been reflected in the fact that ever more production is occurring within value chains, both at home and abroad. Theoretical and empirical approaches to the analysis of value chains have advanced rapidly, yet are very eclectic and heterogeneous. The earliest definitions of commodity chains^1^ date back to the world-systems^2^ theory: “What we mean by such chains is the following: take an ultimate consumable item and trace back the set of inputs that culminated in this item— the prior transformations, the raw materials, the transportation mechanisms, the labour input into each of the material processes, the food inputs into the labour. This linked set of processes we call a commodity chain (Hopkins and Wallerstein 1977)”. In the 1990s, the research programme of global commodity chains was first systematically outlined by Gereffi’s seminal contribution (Gereffi 1994) that defined three interlocking dimensions of the research: the input–output dimension, the spatial dimension, and the question of commodity chain governance^3^. This research period was characterised by moving away from a historical and macroeconomic perspective towards a special focus on industrial chains and the inter-firm cooperation perspective, with numerous case studies on value chains. The global value chain framework emerged early in the new century with the express aim of unifying the previous heterogeneous research (Gereffi 1999; Gereffi et al. 2001). On one hand, the global value chain approach increased the focus on the enterprise level and merged with the literature from international business and management^4^, while also drawing from the new institutional transaction cost approach^5^. On the other hand, the creation of international input–output tables^6^ led to a revival of the aggregated macroeconomic approach to global value chains, albeit with a different focus than the world-systems approach.^7^

In this article, we present a new methodology for measuring different value chain participation rates in the international input–output framework. Compared to the most widely used measurement of value chain participation introduced by Wang et al. (2017), we make two fundamental conceptual enhancements.

First, our methodology creates a single and consistent measurement of value chain participation on the country-sector level, as opposed to the two (upstream and downstream) participation rates that feature in Wang’s methodology. The argumentation and logic used to derive a single value chain participation share on the country-sector level is very similar to the approach of Arto et al. (2019), which combines the source- and sink-based approaches to export decomposition. The idea is that decomposition based on final demand (sink-based decomposition) is independent of the decomposition of downstream value added (source-based) and thus both can be linearly combined to grasp both the information regarding the source of value added as well as the path to final demand simultaneously. Methodologies of export decomposition have recently seen significant improvements (Arto et al. 2019; Borin and Mancini 2019; Miroudot and Ye 2021). However, the value chain participation rate methodologies either still chiefly rely on the value-added export matrix to describe the value flows between any two country-sectors in the economy (Johnson and Noguera 2012) and result in separate upstream and downstream participation rate measures or combine a sink- and a source-based measure in merely one-sided, forward-looking measures. Our approach to value chain decomposition no longer uses the value-added export matrix and instead breaks down the asymmetric value chain stemming both downstream and upstream from each country-sector concerned *simultaneously*. Creating a single consistent variable on the country-sector level that measures the overall level of participation in value chains enables the empirical testing of many research theses that were previously either limited to the aggregate level or had to be articulated separately in terms of measuring the impacts of upstream and downstream value chain integration.

Second, our methodology allows extensions of the value chain typology that are not possible with Wang’s approach to the decomposition of production activities or with export decompositions. We introduce a novel measure of the domestic value chain participation rate to measure the share of production which represents the extent of the fragmentation of domestic production. In place of a single and undifferentiated domestic component, we distinguish domestic production, which is fragmented (involving measurable cooperation among domestic firms), and domestic production, which is not fragmented (consisting of producing direct value for consumption without the cooperation of domestic firms). This makes our concept of the domestic value chain a completely new and different concept compared to Wang’s domestic component, which does not distinguish the two and combines both categories within a single undifferentiated concept. While Wang’s share of the domestic component is only a simple residual—a negation of the share of the fragmentation of global production and the global Ricardian trade share that does not provide information about the nature of the domestic economy, our novel methodology allows us to measure the extent of fragmentation of domestic production in addition to the usual study of the fragmentation of international production.

We aim to use our approach to provide methodological tools that facilitate exploration of the complex interrelationship of global and domestic value chains and their evolution over time. We believe this will add to understanding of the diverse patterns of the structural integration of various countries/sectors and the different effects of such patterns on economic development. While this is primarily a methodological contribution, we shall use elementary empirical data to try to show the possible link between the level of fragmentation of global and domestic production and overall economic growth.

The article is structured as follows: In Sect. 2, we review the existing value chain indicators and address their shortcomings. In Sect. 3, we present our methodology. In Sect. 3.1, we present a new conceptualisation of value chain in the international I–O framework and define our object of disaggregation. A new value chain typology is presented in Sect. 3.2 where we also derive participation shares. In Sect. 4, we present an example of empirical application and some basic empirical results of the new methodology to show the insights into economic structures that can be gained by using the new value chain measures and which links exist between value chain integration patterns and overall economic growth. Finally, we discuss the contributions of the paper, its limitations and possibilities for further research.

## Background

The most recent macroeconomic analyses of global value chains rely on the international input–output methodology. As international I–O data are essentially an integrated standard accounting data set harmonised on the sectoral level, information is lacking on the typology of value chain governance. This means the international I–O database cannot be the sole source for the study of production networks, which theoretically differ from purely open trade transactions by including at least some level of hierarchy, and which investigate the local embedding of production linkages (Buckley 2009; Henderson et al. 2002; Hess and Coe 2006; Hortaçsu and Syverson 2009). However, the general framework of global value chains can function without such distinctions and this makes the international I–O data set one of its most important sources of information. The key benefit of applying the I–O methodology in global value chain analysis is that aggregated information about the structure of value chains can be obtained, as opposed to isolated firm-specific case studies that can provide a more detailed understanding of different aspects of a given value chain. Thus, of the three dimensions of commodity chain research noted by Gereffi (1994), both the I–O aspect and the spatial dimension, can be considered in the international I–O approach, while the governance aspect cannot. Various aggregated and sectoral global value chain indicators, indices and measures have been proposed, all derived from the international I–O framework. GVC indicators may be roughly divided into measures of length^8^ and participation rates, which we will discuss briefly.

Early I–O measures of the GVC structure were simple upstream and downstream indicators that corresponded to the measure of distance to final demand (upstream) and the Leontief measure of backward linkage (downstream) and were often referred to as the length of a value chain (Ahmad et al. 2017). Fally (2011) and Antràs et al. (2012) defined the downstream indicator to “reflect how many plants (stages) are involved in production one after the other” up to the point observed and the upstream indicator to “measure how many plants this product will pass through (e.g. by assembly with other products) before it reaches final demand (Fally 2011, 10)”. Fally (2011) defined them as the number of vertical stages weighted by the value added of each stage, with the distance between each stage set to 1.^9^ Since then, the average vertical distance has been the basic measure of the length of the value chain in the international I–O framework. Miller and Temurshoev (2015) further specified the existing measures by presenting upstream and downstream indicators in a matrix formulation using Ghosh’s forward and Leontief’s backward coefficient matrices (Ghosh 1958; Leontief 1936). These upstream and downstream measures are simple measures of the upstream and downstream length of value chains measured by the average vertical distance. Within this framework, further improvements were introduced by Muradov (2016), who focused on separating the domestic from the global production component while calculating the length of value chains.

The existing dominant conceptualisation of GVC participation measures is largely based on the work of Johnson and Noguera (2012), who produced a value-added export matrix that captures information on value flows in the economy between any two points (country-sectors) in the economy. This provides the basis for the disaggregation of value on the country-sector level, depending on whether the value was produced domestically for domestic consumption or involved cross-border transactions for either final or productive consumption (Koopman et al. 2014; Los et al. 2015; Wang et al. 2017). Since the value-added export matrix tells us about the source and destination of value added and covers all possible paths between any two country-sectors in the economy, there are two indicators of the share of GVC participation—the upstream and downstream share. The conception of the upstream participation share of participation starts from the value added of individual industries (country-sectors), disaggregating all possible paths leading to the realisation of their value, while the conception of the downstream share of participation starts with final consumption, disaggregating all possible paths of the downstream production linkages. Within this framework, disaggregation is defined on the domestic part, the “Ricardian trade” in finished goods, the simple GVC and the complex GVC, which is currently the most widely used accounting framework for GVC participation and thus far has been used by the best-known research on GVC carried out jointly by the WTO, the WB group, the OECD, IDE-JETRO, RCGVC-UIBE and the China Development Research Foundation (GVC Development Reports). Further improvements and clarifications of the framework were made by Borin and Mancini (2019), who derive their own measure of GVC-related bilateral trade flows by decomposing export to that attributable to traditional trade and GVC trade. Their indicator is composed of source-based backward and sink-based forward parts of their export decomposition, which can be calculated in a bilateral, country and world setting.

The development of I–O participation share measures of value chains, which are the primary interest of this article, evolved simultaneously with the development of methodologies of decomposing trade in value added (Johnson and Noguera 2012) as well as value added in trade (Arto et al. 2019; Borin and Mancini 2019; Miroudot and Ye 2021). However, despite similarities and some conceptual and formal mathematical overlapping, the fields of value chain participation share measures and value added in trade are driven by quite distinct research questions and research interests. On one hand, principal interest in decomposing exports is the correct evaluation of cross-border flows (properly removing double counting), assessing trade policy impacts and conducting overall impact analysis, either in a bilateral setting or with a focus on a specific country. On the other hand, value chain participation measures attempt to grasp the structure of an economy, sectoral and country interdependencies and the specific embeddedness of each production unit in different value chain structures, both at home and abroad. Value chain participation share measures usually correspond to a share of production, which statistically satisfies certain *a priori* criteria, such as “at least two cross-border transactions” or “at least one cross-border production sharing transaction”. The reviewed literature has contributed to better understanding of value chains and their I–O applied research, but still suffers two shortcomings that we try to address and improve with our approach.

The first main shortcoming of all current value chain participation share indicators is the lack of a single uniform measure for different value chain participation rates on the country-sector level. First, the value chain decomposition of Wang et al. (2017) results in downstream and upstream value chain participation rates, which provide two different types of information at the country-sector level. This is relevant for some types of analysis that deal with the relationship between upstream and downstream participation in GVCs, but there is a variety of situations where a common measure of GVC participation, defined uniformly on the country-sector level, is required either as the main object of the analysis or as a supplementary or control variable.^10^ Second, GVC measures based on the decomposition of exports, even though they overcome the sink- and source-based decomposition in one unifying framework of export decomposition (Arto et al. 2019; Borin and Mancini 2019), are conceptually unable to offer a consistent solution to the question of a single country-sector value chain participation measure. That is because the criteria for export decomposition (separating domestic value added from foreign value added and the removal of double counting) do not correspond with the general criteria for different value chains on the country-sector level (the share of production with a certain number of cross-border transactions). Although export can be decomposed both with regard to the origin of the value added as well as the final demand, the very fact that the object of decomposition is export means it has a one-sided, forward orientation since export decomposition cannot address the fragmentation of production of a country-sector that has little or no exports (but can still form part of the fragmentation of a global value chain downstream). In this sense, the attempt by Borin and Mancini (2019) to provide a GVC measure of bilateral trade by decomposing exports cannot identify the share of production of a given country-sector which satisfies the criterion of a certain number of cross-border transactions, but only examines its forward part and is hence conceptually similar to Wang’s forward GVC measure. Our attempt to solve this issue demands the decomposition of the gross output (total output) of each country-sector to simultaneously account for both downstream and upstream value chain linkages.

The second major shortcoming of existing value chain indicators is the lack of a measure of domestic value chain fragmentation. The decomposition put forward by Wang et al. (2017) includes a broadly defined “domestic component”, which covers all of the value that does not comply with the GVC and Ricardian trade criteria. One of the major contributions of this article is to conceptually further divide this broad domestic component into a first part which comprises domestic production fragmentation (involving production sharing between at least two domestic firms) and the second part which does not. This yields new information regarding the share of production not involved in the fragmentation of global production, but is part of the fragmentation of domestic production and enables research into the role of domestic production fragmentation, which was impossible with the existing conceptualisations. As a result of the present disaggregation of participation shares into the “domestic component” and the GVC participation rates (and the Ricardian trade share) consisting of a simple duality that in its construction sums to 1, the share of the domestic component is never used in regressions (due to collinearity) and never even examined as a theoretical concept. It is simply a residual, a share that does not interest researchers given that all the information they disaggregate is included in their GVC participation rates. The existing approaches are used by researchers to focus exclusively on the international dimension of the fragmentation of production, neglecting the potential held by the international I–O methodology that allows analysis of domestic production fragmentation. Our approach is breaks ground in this area as it proposes a new concept of domestic fragmentation able to be measured on its own and according to its own definition and that is not collinear with the sum of the GVC participation rate.

**Fig. 1:**
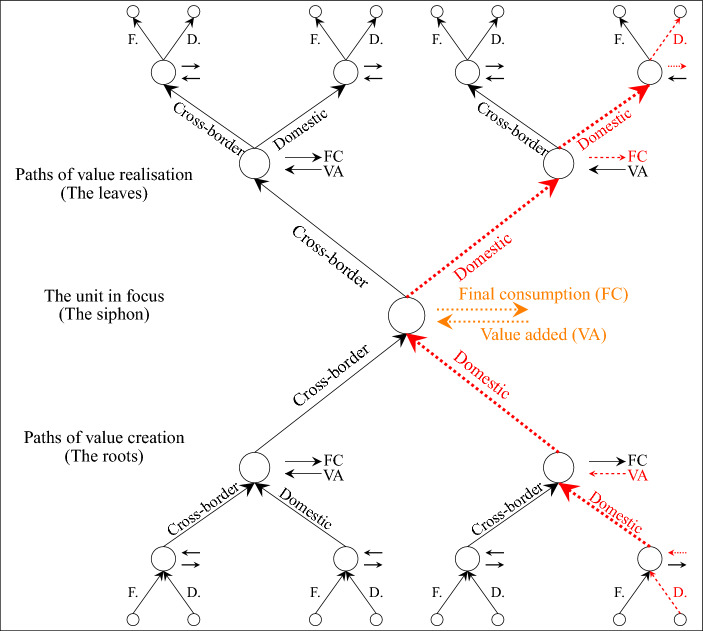
Value chain tree. Source: own conceptualisation and design. Arrows represent production-sharing transactions—buying and selling of intermediate products for production. Orange colour denotes production that does not involve any production sharing, while any combination of red or orange paths denotes domestic production fragmentation. Any value chain path which includes a cross-country production-sharing transaction (a black arrow) is part of a global value chain from the perspective of the particular unit in focus. The paths of value creating and value realisation in a general case continue to branch ad infinitum (three levels are chosen only for demonstration purposes)

Our methodological approach starts with the formal criteria, which is common for most of the GVC literature where value chains are defined according to certain transaction criteria (number of cross-border production-sharing transactions or similar). It is important to note that any such criteria are arbitrary and potential multiplicity of such criteria and hence value chain typologies can coexist and offer researchers some leeway in their empirical applications.^11^ With a view to creating a uniform value chain measure on the country-sector level, we use the total output of each country-sector as the starting point of our disaggregation. Decomposing total output (as opposed to export or total value added) enables us to simultaneously grasp both the downstream and upstream value chain paths as well as the structure of the economy that is entirely domestic. Our decomposition begins with a set of the presented value chain tree matrices ($\tau _i$) which describe all of the value chain paths,* from any *country-sector of primary origin *to any* country-sector of production for final consumption that *passes through* (include a production stage of) *a single *particular country-sector. The logic of our approach is very similar to that of Arto et al. (2019) for combining the sink- and source-based decomposition of exports: because the decomposition of paths to final demand is independent of the decomposition of downstream value added, these decompositions can be linearly combined to capture both types of information in a single decomposition along two different dimensions at the same time. The big distinction with this approach is that object of decomposition is different—in our case, it is the total output (gross output) of each country-sector. Our choice of the object of decomposition is a prerequisite for properly capturing downstream linkages and, more importantly, properly accounting for the domestic structure of the economy. This formulation is the first attempt to capture information concerning the asymmetric value chain tree, which is a specific feature of each individual country-sector (Fig. 1). The proposed value chain tree matrices are unique in that they allow us to simultaneously capture the structure of the downstream and upstream value chain paths and to define value chain participation rates as a single measure for each country-sector. The crucial point of the proposed methodology is to enable the disaggregation of value chains based solely on the structure of value chain paths—taking into account whether these paths include only domestic production fragmentation, international production fragmentation or no production fragmentation at all. This allows us to introduce the concept of domestic value chain fragmentation that simply cannot be created within the existing framework of 2 separate participation indices. This multiplies the research opportunities offered by the value chain methodology based on the international input–output structure by permitting general analysis of the fragmentation of both domestic and global production and their interdependence along with any mutual effects of their development.

Applying this methodology, we show that increasing fragmentation of global production in recent decades has been a general trend for most countries (with a backlash in later years), but different institutional arrangements and structural economic positions led to various types of global economic integration, bringing diverse effects for domestic fragmentation. With our methodology, we shall empirically demonstrate that in many countries with high growth and ever stronger global integration domestic fragmentation also increased. However, one can find cases where domestic fragmentation stagnated or even declined whereas fragmentation of the global value chain increased. The different types of integration in global value chains are the outcome of several structural and institutional developments.^12^ On one hand, the simultaneous increase in domestic and global fragmentation might only be a consequence of the growing complexity and division of labour. Yet, on the other hand, the simultaneous rise in global fragmentation and drastic decline in domestic integration might be due to the fracturing of domestic vertically integrated companies, parts of which are integrated into global value chains as subsidiaries, or due to the gradual replacement of domestic suppliers by globally traded inputs, which may increase following a foreign takeover or privatisation. The wide range of possibilities mean that every production unit may hold a different structural position within global production as a whole, and different structural positions may imply varying levels of dependence, which can be a factor of economic performance, especially during a crisis (Horvath and Grabowski 1999).

## Methods

### The value chain tree

#### Conceptualisation

We understand a value chain as a series of stages in the production of a product or service for the end user, where each stage adds value and the total value of the end product is the sum of the value added in each stage. For a value chain to exist, there must be at least two separate production stages. The existing GVC framework is analytically and empirically based on the idea that value is created in the production process and added to the value already present in the intermediate goods being used. The old value (value of intermediaries) is only transferred to the new product, while the newly created value is added linearly to the transferred value. The same idea also lies behind the elimination of double counting in standard gross trade statistics and exploration of the hidden underlying trade in value added, which provides insight into the international structure of trade (Arto et al. 2019; Johnson and Noguera 2012; Miroudot and Ye 2021). We make the same basic assumptions for value chain analysis.

We examine the structure of the economy from the perspective of a small unit^13^ (country-sector) and capture its structural position within domestic and international production by measuring the degree of integration into domestic or global value chains. Each production unit is located within the production structure with a number of production-sharing transactions. On one side, the conditions of production are linked to the inputs produced by other firms in downstream linkages and, on the other, the final consumption of its product may only be reached after a series of upstream linkages in which its output is used as an input by other firms.

Accordingly, if one concentrates on a specific unit (country-sector) and aims to capture the upstream and downstream value chain linkages *simultaneously*, the value chain can be viewed as a tree, in contrast to the snake or spider analogy (see Fig. 1).^14^ In the general case, the product is partly consumed immediately after production but also partly sent on to further stages of production and from each of these upstream stages it is further decomposed in the same way (etc., ad infinitum), spreading out like twigs and leaves until it ends completely in final consumption. Similarly, the primary value-creating activity can be represented by the structure of the roots, whereby value is only partially created in each stage since it requires pre-existing intermediates, which in turn are further decomposed in the same way ad infinitum.

To conceptualise and measure the value chain structure of each specific smallest unit of analysis (country-sector), we introduce the value chain path concept. From the perspective of a firm, a value chain path is a series of transactions between firms that lead from a value-adding process to final demand. While currently no data exist that would account for every transaction between all firms^15^, firm transactions still represent a basis for any I–O sectoral aggregation, which can help us detect tangible differences in the value chain path structure in different country-sectors. While it is impossible with the given limits of accounting data to follow a certain value chain path of each specific product of each specific firm, it is nevertheless possible to analyse the average sectoral structure of value chain paths subject to whether the aggregated transactions between firms (and to the final consumer) are domestic or global. Our use of the signifier “transactions between firms” and “production-sharing transactions” thus does not refer to individual transactions, but instead refers to the information captured by the aggregated sectoral international I–O data regarding the average structure of value chain transactions. Since we do not focus on following transactions for an individual product but distinguish domestic from cross-border transactions between production units, aggregated I–O data are a sufficient starting point. While the accounting rules require transactions between firms in the same sector and the same country to be formally accounted (represented in aggregated form by the purely diagonal elements of the international Leontief coefficient matrix), the same goes for transactions between domestic firms from different sectors (represented in aggregated form by the block diagonal elements of the international Leontief coefficient matrix with purely diagonal elements 0). In this aggregated setting, one can differentiate between domestic and cross-border transactions (quantitatively in terms of shares), which gives the basis for decomposing different value chain paths based on the criterion of the number of cross-border or domestic production-sharing transactions. As shown in Fig. 1, the value chain path can be decomposed with respect to two dimensions: Origin (where the value was primarily created) and the final stage of production (where the end product for consumption is finished).

Our goal of deriving a single value chain participation share measure on the country-sector level requires the derivation of an object able to track the value passing through a specific country-sector in focus along all possible paths from its origin to its end use. In this way, we decompose the value that forms part of the production process of a given country-sector along all its paths, which not only include the downstream paths leading to the country-sector under study and the upstream paths leading from it to final consumption, but also, and above all, the paths that combine upstream and downstream linkages and pass through that country-sector. In general, any value share can originate in any country-sector, and the same value share can also reach final consumption as a product of any country-sector. Compared to the approach of Johnson and Noguera, we add a third dimension^16^—the midpoint—the siphon through which the value from any origin to any final stage flows (Fig. 1), by combining decompositions based on value added and the final demand value chain path. This approach relies on similar reasoning as that of decomposing exports based on both value added and final demand (Arto et al. 2019).

The value chain tree of each country-sector is defined as the structure of the value chain paths, where this country-sector is the siphon via which the value chain paths pass. We show that each unit of analysis (country-sector) has a unique value chain structure that represents its structural position in the economy. Its output can be decomposed along every possible path within its value chain tree—i.e. along every value chain path that has its primary origin in any country-sector, passes through downstream linkages to the production stage of the country-sector which defines the value chain tree (the siphon), and ends in final consumption through upstream linkages as the final product of any country-sector.

Understanding the structure of value chains by empirically measuring all such paths of each country-sector (the smallest unit of analysis) is already an end in itself and can help with further understanding of the economy and its changing structure in terms of global integration, its specific regional and sectoral forms, and the complex interactions between domestic and global production fragmentation.

#### Derivation

The object of disaggregation is a country-sector’s total output. Each country-sector’s total output is disaggregated along both downstream and upstream linkages that are unique to its specific value chain structure. Downstream disaggregation represents all possible value chain paths from the origin of production and upstream disaggregation all possible paths to satisfy the final demand, both with respect to the unique value chain tree of each country-sector. In this way, we disaggregate the same object—the total output of each country-sector—simultaneously along its downstream and upstream paths.

In contrast to approaches based on the matrix of value-added exports (Johnson and Noguera 2012; Wang et al. 2017) to cover all value-added flows between any two country-sectors in an economy, we propose a new object—a set of matrices that describe the value chain structure of each country-sector separately, covering all value chain paths from each primary origin to each final stage via the output of a single specific country-sector (Fig. 1). In this conceptualisation, each country-sector has a corresponding value chain tree described by the value chain tree matrix—while the value chain structure of the economy as a whole is described by the set of such matrices.

We derive our disaggregation within the static international Leontief demand-driven model. *C*, *F* and *x* are the main accounting datasets representing the intermediate consumption matrix, final consumption matrix and total output vector. The Leontief coefficient matrix is usually derived as $A=C{\hat{x}} ^{-1}$. The variables with hat are vectors transformed into diagonal matrices, ${\hat{f}}$ represents a diagonal matrix of final demand and ${\hat{v}}_C$ a diagonal matrix of value-added coefficients.^17^ The usual pairs of indices characterising the country and sector of origin (*s*,* i*) and the final destination (*d*,* j*) are replaced by a single index for each country-sector for more transparent notation. Since we are no longer working in the $n\times n$ dimensional space, but in the $n \times n \times n$ dimensional space, we would need 3 pairs of indices, 1 pair for the country-sector of origin, 1 pair for the final stage and also 1 pair for the country-sector, which is the siphon through which all possible value chain paths characterise its specific value chain structure. Instead, we are working with only 3 indices, one for the country-sector of origin (*k*), one for the final stage country-sector (*j*) and one to characterise the country-sector value chain tree—the country-sector representing the siphon through which the value chain paths pass (*i*).^18^

3.1 $$\begin{aligned} x= & {} C{\mathbf {1}}+F\vec {1} \end{aligned}$$

3.2 $$\begin{aligned} x= & {} Ax+f \end{aligned}$$

We start with the upstream part, by using standard Leontief’s derivation:

3.3 $$\begin{aligned} x= & {} (I-A)^{-1}f, \end{aligned}$$

3.4 $$\begin{aligned} x= & {} (I-A)^{-1}{\hat{f}}{\mathbf {1}}, \end{aligned}$$

3.5 $$\begin{aligned} {\hat{x}}^{-1}(I-A)^{-1}{\hat{f}}{\mathbf {1}}= & {} {\mathbf {1}}. \end{aligned}$$

##### Definition 1

Upstream output decomposition *W*:

$$W={\hat{x}}^{-1}(I-A)^{-1}{\hat{f}}.$$

The matrix *W* represents the upstream output decomposition along all upstream value chain paths. Its element $w_{ij}$ represents the share of the total output of country-sector *i* that reaches final consumption as the end product of country-sector *j*, along all possible upstream production fragmentation paths in the economy. The *i*th row of *W* represents the disaggregation of the total output of the *i*th country-sector into output shares according to its final production stages that account for all direct and indirect paths of the upstream value transfers leading to the full realisation of total output (by being used directly or indirectly by other country-sectors as intermediate productive consumption). Each *i*th row of *W* may thus be characterised as a discrete probability distribution. On one hand, the upstream output shares of each country-sector *i* add up consistently to 1: $\sum _{j=1}^n w_{ij}=1$ $\forall i$. On the other hand, there is a clear economic interpretation of the probability distribution: $w_{ij}$ represents the probability that a randomly selected part of the total output of the *i*th country-sector will eventually be consumed as the final product of country-sector *j*, along any upstream value chain path.

For the downstream part, we begin with identity:

3.6 $$\begin{aligned}&x^T={\mathbf {1}}^T{\hat{x}}, \end{aligned}$$

3.7 $$\begin{aligned}&x^T={\mathbf {1}}^T(I-A)(I-A)^{-1}{\hat{x}}, \end{aligned}$$

3.8 $$\begin{aligned}&x^T=v_C^T(I-A)^{-1}{\hat{x}}, \end{aligned}$$

3.9 $$\begin{aligned}&x^T={\mathbf {1}}^T \hat{v_C} (I-A)^{-1}{\hat{x}}, \end{aligned}$$

3.10 $$\begin{aligned}&{\mathbf {1}}^T={\mathbf {1}}^T \hat{v_C} (I-A)^{-1}. \end{aligned}$$

##### Definition 2

Downstream output decomposition *Z*:

$$Z= \hat{v_C} (I-A)^{-1}.$$

The matrix *Z* represents the downstream output decomposition along all downstream value chain paths. Its element $z_{ki}$ represents the share of the total output of country-sector *i* that is primarily created in country-sector *k*, along any possible downstream production fragmentation path in the economy. The *i*th column of *Z* represents the disaggregation of the total output of the *i*th country-sector into output shares, which represent all direct and indirect paths of the downstream value transfer from each country-sector that has contributed to the production of its output (through the direct or indirect production of intermediate productive consumption used by *i*). Each *i*th column of *Z* may thus be characterised as a discrete probability distribution. On one hand, the downstream output shares of the individual country-sectors *i* add up consistently to 1: $\sum _{k=1}^n z_{ki}=1$ $\forall i$. On the other hand, there is a clear economic interpretation of the probability distribution: $z_{ki}$ represents the probability that a randomly selected part of the total output of the *i*th country-sector was produced by country-sector *k*, along any downstream value chain path.

The two matrices presented, *W* and *Z*, may appear as two sides of the same coin—similar to forward and backward decomposition, which has largely been exhausted in the international input–output literature. However, if we focus on a single country-sector (*i*), the *i*th column of *Z* and the *i*th row of *W* represent two probability distributions that take the transfers in the value chain into account, which result in two completely different and independent types of information. The *i*th column of *Z* contains information on the downstream structure of the value chain of the respective *i*th country-sector and the *i*th row of *W* contains information on the upstream structure of the value chain of the respective *i*th country-sector. For a given *i*th country-sector, the two probability distributions are asymmetrical. Most importantly, both probability distributions relate to the same object of investigation—the total output of country-sector *i*.

Using the total output of each country-sector seems to be the only way to disaggregate the same object into its upstream and downstream value chains. The object of decomposition of the upstream part (which is decomposed based on the paths to final demand) of a certain country-sector can be either its total output or total value added (even its export). However, the same is not possible for the downstream part (which is decomposed according to the origins of its value-added). The object of decomposition of the downstream part of a certain country-sector can only be its total output, which also makes up the totality of value-added shares along the whole downstream value chain.^19^ In other words, the country-sector’s total output is an object that has both an upstream and a downstream path, while total value added and total export represent only that part of the output which has an upstream path, even if this upstream path is disaggregated by value-added origin. Using the total output share as the basis for disaggregating to the individual country-sector level is therefore a legitimate choice. This mainly explains why we derived the *W* matrix in terms of shares of total output (3.4, 3.5) and not, as is usual, in terms of shares of value added—to make it perfectly clear that both upstream and downstream disaggregation have the same object—the total output of *i*, which includes both the value added of country-sector *i* and the total value added of the other country-sectors (*k*) downstream. The same object (total output) is then distributed along the upstream value chain paths (as determined by the *i*th row of *W*) until it reaches final consumption along an upstream value chain path.

All input–output analyses assume the homogeneity of the smallest classification object (country-sector in our case). The level of detail of the data corresponds to the level of detail of the sector (and country) classification and within a country-sector there is no further information and quite strict homogeneity assumptions apply. We use the assumption of the homogeneity of production of each country-sector to combine the two probability distributions.

$z_{ki}$ represents the share of the total output of the *i*th country-sector, which was primarily produced by country-sector *k*. Due to the homogeneity of the total output of the *i*th country-sector, the $w_{ij}$ represents not only the probability that a random part of the total output of the *i*th country-sector reaches final consumption as a product of *j*, but also the probability that a random part of any share of the output of the *i*th country-sector reaches final consumption as a product of *j*. Since $z_{ki}$ is a share of the *i*th country-sector’s total output, its upstream decomposition is clearly and uniquely defined by the *i*th row of *w*.

The product $w_{ij}z_{ki}$ thus simply represents the probability that a certain part of the total output of the *i*th country-sector is primarily produced in *k* and reaches final consumption as the product of *j* along any value chain path (upstream, downstream or a combination) passing through *i*. In other words, it represents the share of the total output of *i* that was produced by *k* and reached final consumption as a product of *j*. A simple multiplication of probabilities requires that the two events—a random portion of the total output of *i* produced by *k* and a random portion of the total output of *i* completed for consumption by *j*—are statistically independent. First, if certain parts of the total output of a particular country-sector were to behave differently from certain other parts of the same output, this would violate the homogeneity assumption, which is the basic assumption of the input–output structure and methodology. Second, at the level of economic theory it is relatively easy to argue about the statistical independence of the structure of upstream and downstream value chains: Nothing about the downstream structure of production in the *i*th country-sector implies anything about its upstream structure and *vice versa*. Both are calculated independently and provide completely different information: the downstream decomposition gives information about the inputs produced by other country-sectors used directly or indirectly in the production process of the *i*th country-sector, and the upstream decomposition gives information about how the product of the *i*th country-sector is consumed either directly or as part of the final product of other country-sectors.

Two separate vectors which disaggregate the value chain paths of the downstream (*i*th column of *Z*) and upstream value chain (*i*th row of *W*) thus span an entire matrix of total output shares that capture the value chain tree structure of the *i*th country-sector. We combine them with the direct product that defines the matrix of the value chain tree for each country-sector (*i*) by multiplying each element of $Z\vec {e_i}$ (the *i*th column of *Z*) by each element of $\vec {e_i}^{T}W$ (the *i*th row of *W*).

##### Definition 3

Value chain tree matrix

$\tau _i=Z\vec {e_i} \otimes \vec {e_i}^{T}W$; $\tau _i\in \mathbb{R}^{n\times n}$, where $\vec {e_i} \in \mathbb{R}^n$ represents the standard orthonormal basis of $\mathbb{R}^n.$

This defines each element of the value chain tree matrix $t_{ijk} \in \tau _i$ as $t_{ijk}=w_{ij}z_{ki}$. Each element of the value chain tree matrix $\tau _i$ thus represents a share of the total output of country-sector *i*, which is primarily produced in country-sector *k* and consumed as an end product of country-sector *j*, along any upstream and downstream value chain path.

The main point of our derivation is not the expressed final value distribution of the total output of each country-sector along any of its upstream and downstream value chain paths, but the expression of the total output distribution (of the respective country-sector) along any value chain path, be it a downstream value chain path, an upstream value chain path or any combination of both paths at the same time.

3.11 $$\begin{aligned} \tau _i=\hat{v_C}(I-A)^{-1}\vec {e_i} \otimes \vec {e_i}^{T}{\hat{x}}^{-1}(I-A)^{-1}{\hat{f}} \end{aligned}$$

The structure of the value chain tree matrices allows us to focus our disaggregation on the composition of the value chain paths covered by the two global Leontief inverses in the equation, the first representing all downstream parts of the value chain paths and the second representing all upstream parts of the value chain paths.

A single value chain path is determined by a series of concrete transactions between companies: It is a unique path from primary value creation (value created in production, not transferred from intermediate products) to value realisation (final consumption, not productive consumption of intermediate products), which passes through the production stage of the *i*th country-sector. The total output of *i* is not only disaggregated along all possible paths leading from any country-sector of origin via country-sector *i* to any country-sector of final stage production (as determined by $\tau _i$), but is also disaggregated in much finer detail, along all the unique value chain paths that pass through *i*. That a concrete value chain path only forms part of the value chain tree matrix can easily be recognised if both inverses in $\tau _i$ are replaced by an infinite series ($(I-A)^{-1}=I+A+A^2+\cdots$). Such disaggregation then results in an infinite number of value chain paths, and the total output of the *i*th country-sector is distributed over all of these paths.

A certain value chain path share of the total output of *i* is determined by the Leontief technical coefficients $a_{ij}\in A$. For example, take a value chain path consisting of value primarily produced in country-sector $CS_1$^20^, then used as an intermediate in $CS_2$, which in turn is used as an intermediate in *i* (the country-sector whose value chain is broken down), and then sent as an intermediate to $CS_3$, which is then sent as an intermediate to $CS_4$, where it is finished and sold for consumption. This value chain path has an origin ($CS_1$), a midpoint (*i*) and a final destination of production ($CS_4$), as well as a concrete path with a length of 5 (5 country-sectors contribute to production from origin to final consumption). The share of the total output of the *i*th country-sector that may be attributed to this specific path is:

3.12 $$\begin{aligned} v_{CS_1}a_{CS_1CS_2}a_{CS_2 i}x^{-1}_ia_{iCS_3}a_{CS_3CS_4}f_{CS_4}. \end{aligned}$$

A specific unique value chain path of the *i*th country-sector’s value chain tree, that has its origin in *k* and final stage in *j*, can be written as:

3.13 $$\begin{aligned} \Pi _{p=1}^{d} v_{CS_0}a_{CS_{p-1}CS_{p}} x^{-1}_i \Pi _{p=d}^{u-1}a_{CS_{p}CS_{p+1}} f_{CS_u}. \end{aligned}$$

Such a path has a downstream length of *d* and an upstream length of $u-1-d$ and the path is determined by a unique set of production-sharing transactions from the origin to the final stage (from origin $j=CS_0$, to $CS_1$, to $CS_2$, ..., to $i=CS_{d}$, and further to $CS_{d+1}$, $CS_{d+2}$, ..., to $k=CS_u$). Leontief technical coefficients $a_{CS_{p-1}CS_{p}}$ determine each production-sharing transaction. The summation along the total output shares of *i* attributed to all such unique value chain paths, taking into account all permutations of possible transaction sequences and also all possible lengths (all possible length combinations of downstream and upstream lengths) as well as all possible origins and final stage destinations, results in a unit:

3.14 $$\begin{aligned} {\mathbf {1}}^T \tau _i {\mathbf {1}}= {\mathbf {1}}^T\hat{v_C}(I-A)^{-1}\vec {e_i} \otimes \vec {e_i}^{T}{\hat{x}}^{-1}(I-A)^{-1}{\hat{f}} {\mathbf {1}}^T = 1. \end{aligned}$$

Our conceptualisation allows us to define decomposition criteria applicable to each value chain path of the value chain tree of the *i*th country-sector. Based on this property, we will decompose the value chain structure of each country-sector separately in the following section.

### The value chain typology

#### Definitions

The framework of the international I–O analysis allows the separate analysis of final transactions to consumers and transactions between companies. Based on this characteristic, we propose a typology of value chains based solely on the structure of linkages between enterprises, while adding a further decomposition with regard to different possible transactions to reach the final consumption *post festum*.^21^ Each matrix $\tau _i$ expressed by equation 3.13 represents the desegmentation of the total product of country-sector *i* along different downstream and upstream paths. When we refer to a value chain, we refer to the specific share of value (share of output) that corresponds to a particular value chain path. Path^22^ of each value share generally includes any combination of domestic and cross-border production-sharing transactions, which can take place both downstream and upstream relative to the respective country-sector. Our criteria for the value chain typology thus refer to each specific value share corresponding to a single path within a value chain tree specific to each country-sector.

##### Definition 4

Domestic value chain

Domestic value chain (DVC) is a value that involves* at least 1 domestic production-sharing transaction* and involves *only domestic production-sharing transactions* along its path.

##### Definition 5

Global value chain

Global value chain (GVC) is a value that involves* at least 1 cross-border production-sharing transaction* along its path. We further distinguish two types of global value chains: simple and complex.

##### Definition 5.1

Simple global value chain

Simple global value chain (SGVC) is a value that involves *exactly 1 cross-border production-sharing transaction* anywhere along its path.

##### Definition 5.2

Complex global value chain

Complex global value chain (CGVC) is a value that involves* more than 1 cross-border production-sharing transaction* along its path.

##### Definition 6

No value chain

No value chain (NVC) is a value that *does not involve any production-sharing transactions* and has no value chain path within production.

A few brief comments are appropriate on our definitions and their interpretation. No material product or service belongs to a single classification of value chain, and no enterprise can be considered part of a single type of value chain. The output of each enterprise belongs to a variety of value chain paths. In general, one part of the output comprises many cross-border transactions, another part only domestic transactions, and yet another part their relatively complex interrelationship. Each product (or country-sector in our case) can be assigned different shares of the value chain paths. These shares are objects that provide information about the structure of the economy. For example, virtually no enterprise could be classified exclusively as part of a no value chain, but some enterprises that provide services (e.g. domestic services) have a relatively high share of output that has no value chain path, especially in services, where salaries account for almost all of the enterprise’s expenditure and where their product directly satisfies final demand. On one hand, enterprises that specialise in intermediate goods are always part of a value chain, whether domestic or global. On the other hand, even modern industries such as food-processing and pharmaceuticals, also have a certain (usually small) share of value added that is not part of any value chain (no value chain share), corresponding to the share of domestic value added in these industries that is also directly consumed (part of output that has no value chain path). The value chain shares and their changes are the object that provide information about the structure of the economy, whether on the sector or country level. As the economy develops, the division of labour also increases, which corresponds to the growing fragmentation of production, in particular international production fragmentation, and a decrease in shares where there is limited or no value chain fragmentation. Compared to the existing typology of value chains, this revised typology allows for analysis of the relationship between global and domestic fragmentation, which might prove especially relevant for the policies of developing countries.

#### The decomposition of paths

Our value chain typology is established according to criteria along the entire value chain. For this reason, we disaggregate the value chain tree matrices $\tau _i$ in terms of criteria for different types of value chain paths. Our decomposition consists of the decomposition of two Leontief inverses, which may be interpreted as the decomposition of the downstream part and upstream part of each value chain path, as defined by equation 3.11: $\tau _i=\hat{v_C}(I-A)^{-1}\vec {e_i} \otimes \vec {e_i}^{T}{\hat{x}}^{-1}(I-A)^{-1}{\hat{f}}$. The decomposition is constructed based on of the criteria of the number of cross-border and domestic production-sharing transactions that are consistent with the revised value chain typology.

First, we investigate the decomposition of only a single Leontief inverse (interpreted symmetrically with respect to our criteria in the upstream and downstream value chain) and only then do we analyse the decomposition of all value chain paths characterised by the two Leontief inverses. The international I–O data have a specific block matrix structure in which the block diagonal elements represent domestic production-sharing transactions and the block off-diagonal elements represent international production-sharing transactions ($A_D$ denotes domestic—block diagonal—and $A_{ CB }$ cross-border—block-off diagonal—part of *A*), which allows us to decompose the Leontief inverse in the following way:

3.15 $$\begin{aligned} \begin{aligned} (I-A)^{-1}&=(I-A_D)^{-1}+(I-A)^{-1}-(I-A_D)^{-1}\\&=I+A_D+A_D^2+A_D^3+ \dots +(I-A)^{-1}-(I-A_D)^{-1}\\&=I+A_D(I+A_D+A_D^2+\dots )+(I-A)^{-1}-(I-A_D)^{-1}\\&=I+A_D(I-A_D)^{-1}+(I-A)^{-1}-(I-A_D)^{-1}\\&=\underbrace{I}_\text {1.)}+\underbrace{A_D(I-A_D)^{-1}}_\text {2.)} +\underbrace{(I-A_D)^{-1}A_{CB}(I-A_D)^{-1}}_\text {3.)}\\&\quad +\underbrace{(I-A)^{-1}-(I-A_D)^{-1}-(I-A_D)^{-1}A_{CB}(I -A_D)^{-1}}_\text {4.)} \end{aligned} \end{aligned}$$

1. *I* obviously represents that part of the output which contains *no production-sharing transactions*—no value chain linkages. In the upstream part, it represents the share of total output that directly satisfies final demand (i.e. no upstream value chain), while in the downstream part it represents the direct value added of the country-sector whose production is being decomposed (i.e. no downstream value chain).
2. $A_D(I-A_D)^{-1}=A_D+A_D^2+A_D^3+ \dots$ represents that part of output which contains* at least 1 domestic* production-sharing transaction and contains *only domestic production-sharing transactions*.
3. $(I-A_D)^{-1}A_{CB}(I-A_D)^{-1}$ represents that part of the output which contains *at least 1 production-sharing transaction* and contains *exactly one cross-border production-sharing transaction* somewhere along its value chain path. This can be demonstrated by paraphrasing the part as all possible combinations of a single cross-border transaction among any possible set of domestic production-sharing transactions that occur before or after the single cross-border production-sharing transaction:
  $$\begin{aligned} (I-A_D)^{-1}A_{CB}(I-A_D)^{-1} &= A_{CB}+A_{CB}{A_D}+A_{CB}{A_D^2}\cdots \\ &\quad +{A_D}A_{CB}+{A_D}A_{CB}{A_D}+{A_D}A_{CB}{A_D^2}\cdots \\ &\quad +{A_D^2} A_{CB}+{A_D^2}A_{CB}{A_D}+{A_D^2}A_{CB}{A_D^2}+\cdots + \\ \vdots \end{aligned}$$
4. $(I-A)^{-1}-(I-A_D)^{-1}-(I-A_D)^{-1}A_{CB}(I-A_D)^{-1}$ represents that part of the output which contains *at least two or more production-sharing transactions*, of which *at least two are cross-border production-sharing transactions*. This logically follows from the fact that parts (1), (2) and (3) cover the total output that contains less than two cross-border transactions, and that the full Leontief inverse covers the total output.

#### Value chain tree matrix decomposition

We proceed by disaggregating all of the value chain paths as they are structured in the value chain tree matrices. Using the decomposition of the Leontief inverse that we disaggregated in the previous subsection and inserting it into Eq. 3.11, we obtain 16 components ($4 \times 4$ product) for each matrix $\tau _i$.^23^ This disaggregation along both the upstream and downstream paths is the basis for deriving value chain shares that correspond to our typology. We decompose each $\tau _i$ matrix describing all possible value chain paths of the output of the *i*th country-sector into a matrix consisting of domestic value chain paths only, a matrix containing all possible global value chain paths (as well as simple and complex global value chain paths separately), and a matrix consisting only of the value that has no value chain path.

##### Definition 7

Domestic value chain tree $\tau _{i}^{DVC}$

$$\begin{aligned} \tau _{i}^{DVC}=&\, \hat{v_C}A_D(I-A_D)^{-1}\vec {e_i} \otimes \vec {e_i}^{T}{\hat{x}}^{-1}{\hat{f}}\nonumber + \hat{v_C}\vec {e_i} \otimes \vec {e_i}^{T}{\hat{x}}^{-1}A_D(I-A_D)^{-1}{\hat{f}}\\&+\hat{v_C}A_D(I-A_D)^{-1}\vec {e_i} \otimes \vec {e_i}^{T}{\hat{x}}^{-1}A_D(I-A_D)^{-1}{\hat{f}}. \end{aligned}$$

The domestic value chain tree represents all value chain paths of the output of each country-sector which, according to Definition 4, are part of the domestic value chains. In Fig. 1, the domestic value chain paths are marked in red. Domestic value chain paths are defined as all paths that contain at least one red-coloured linkage (representing transactions between domestic enterprises) and include only red-coloured linkages and orange paths (representing the value creation or realisation in the respective country-sector in focus). The first part ($\hat{v_C}A_D(I-A_D)^{-1}\vec {e_i} \otimes \vec {e_i}^{T}{\hat{x}}^{-1}{\hat{f}}$) covers the downstream domestic value added (downstream domestic path), which ends as the *i*th country-sector final stage (no upstream path), the second part ($\hat{v_C}\vec {e_i} \otimes \vec {e_i}^{T}{\hat{x}}^{-1}A_D(I-A_D)^{-1}{\hat{f}}$) covers the value added of the *i*th country-sector (no downstream path) that is transferred via the upstream domestic value chain (upstream domestic path), and the third part ($\hat{v_C}A_D(I-A_D)^{-1}\vec {e_i} \otimes \vec {e_i}^{T}{\hat{x}}^{-1}A_D(I-A_D)^{-1}{\hat{f}}$) comprises the downstream domestic value added that is used as an intermediate product in the production of *i* and then used as an intermediary further in the upstream domestic value chain until it reaches final demand (both downstream and upstream domestic paths). All three cases meet the definition of a domestic value chain.

##### Definition 8

Global value chain tree $\tau _{i}^{GVC}$

$$\begin{aligned} \tau _{i}^{GVC}=&\, \hat{v_C}(I-A_D)^{-1}\vec {e_i} \otimes \vec {e_i}^{T}{\hat{x}}^{-1}\big [(I-A)^{-1}-(I-A_D)^{-1}\big ]{\hat{f}} \\&+\hat{v_C}\big [(I-A)^{-1}-(I-A_D)^{-1}\big ]\vec {e_i} \otimes \vec {e_i}^{T}{\hat{x}}^{-1}(I-A_D)^{-1}{\hat{f}} \\&+\hat{v_C}\big [(I-A)^{-1}-(I-A_D)^{-1})\vec {e_i} \otimes \vec {e_i}^{T}{\hat{x}}^{-1}\big [(I-A)^{-1}-(I-A_D)^{-1}\big ]{\hat{f}}.&\end{aligned}$$

The global value chain tree represents all paths of the output of the individual country-sector, which form part of global value chains according to Definition 5. In Fig. 1, the global value chain paths are represented by all paths containing at least one black-coloured linkage (representing cross-border transactions between enterprises). Global value chain paths can contain any number of red (domestic) and orange (no value chain) linkages provided there is at least one black (cross-border) linkage along their path. The first element ($\hat{v_C}(I-A_D)^{-1}\vec {e_i} \otimes \vec {e_i}^{T}{\hat{x}}^{-1}\big [(I-A)^{-1}-(I-A_D)^{-1}\big ]{\hat{f}}$) covers the downstream domestic and no value chain paths, which have global upstream linkages (simple or complex), the second element ($\hat{v_C}\big [(I-A)^{-1}-(I-A_D)^{-1}\big ]\vec {e_i} \otimes \vec {e_i}^{T}{\hat{x}}^{-1}(I-A_D)^{-1}{\hat{f}}$) covers downstream global linkages (simple or complex), which have a upstream domestic or no value chain path and the third element ($\hat{v_C}\big [(I-A)^{-1}-(I-A_D)^{-1}\big ]\vec {e_i} \otimes \vec {e_i}^{T}{\hat{x}}^{-1}\big [(I-A)^{-1}-(I-A_D)^{-1}\big ]{\hat{f}}$) covers the value that has global paths both upstream and downstream. All of these cases correspond to our definition of a global value chain.

##### Definition 8.1

Simple global value chain tree $\tau _{i}^{SGVC}$

$$\begin{aligned} \tau _{i}^{SGVC}=&\,\hat{v_C}(I-A_D)^{-1}\vec {e_i} \otimes \vec {e_i}^{T}{\hat{x}}^{-1}(I-A_D)^{-1}A_{CB}(I-A_D)^{-1}{\hat{f}} \\&+\hat{v_C}(I-A_D)^{-1}A_{CB}(I-A_D)^{-1}\vec {e_i} \otimes \vec {e_i}^{T}{\hat{x}}^{-1}(I-A_D)^{-1}{\hat{f}}. \end{aligned}$$

The simple global value chain tree represents all paths of the output of each country-sector that are part of simple global value chains as defined by 5.1 The first element ($\hat{v_C}(I-A_D)^{-1}\vec {e_i} \otimes \vec {e_i}^{T}{\hat{x}}^{-1}(I-A_D)^{-1}A_{ CB }(I-A_D)^{-1}{\hat{f}}$) covers a downstream domestic and no value chain path that has simple global upstream linkages and the second element ($\hat{v_C}(I-A_D)^{-1}A_{ CB }(I-A_D)^{-1}\vec {e_i} \otimes \vec {e_i}^{T}{\hat{x}}^{-1}(I-A_D)^{-1}{\hat{f}}$) covers downstream simple global linkages that have an upstream domestic or no value chain path. These are the only cases that fit our definition of a simple global value chain. A value chain path covering both downstream and upstream simple global linkages already has more than 1 cross-border transaction and is hence part of a complex global value chain.

##### Definition 8.2

Complex global value chain tree $\tau _{i}^{CGVC}$

$$\begin{aligned} \tau _{i}^{CGVC}=&\, \hat{v_C}(I-A_D)^{-1}\vec {e_i} \otimes \vec {e_i}^{T}{\hat{x}}^{-1}\big [(I-A)^{-1} -(I-A_D)^{-1}-(I-A_D)^{-1}A_{CB}(I-A_D)^{-1}\big ]{\hat{f}} \\&+\hat{v_C}\big [(I-A)^{-1}-(I-A_D)^{-1}-(I-A_D)^{-1}A_{CB}(I-A_D)^{-1} \big ]\vec {e_i} \otimes \vec {e_i}^{T}{\hat{x}}^{-1}(I-A_D)^{-1}{\hat{f}} \\&+\hat{v_C}\big [(I-A)^{-1}-(I-A_D)^{-1}\big ]\vec {e_i} \otimes \vec {e_i}^{T}{\hat{x}}^{-1}\big [(I-A)^{-1}-(I-A_D)^{-1}\big ]{\hat{f}}. \end{aligned}$$

The complex global value chain tree represents all paths of the output of individual country-sectors that form part of complex global value chains as defined in 5.2 The first element ($\hat{v_C}(I-A_D)^{-1}\vec {e_i} \otimes \vec {e_i}^{T}{\hat{x}}^{-1}\big [(I-A)^{-1}-(I-A_D)^{-1}-(I-A_D)^{-1}A_{ CB }(I-A_D)^{-1}\big ]{\hat{f}}$) covers the downstream domestic and no value chain path, having complex global upstream linkages, the second element ($\hat{v_C}\big [(I-A)^{-1}-(I-A_D)^{-1}-(I-A_D)^{-1}A_{ CB }(I-A_D)^{-1}\big ]\vec {e_i} \otimes \vec {e_i}^{T}{\hat{x}}^{-1}(I-A_D)^{-1}{\hat{f}}$) comprises downstream complex global linkages, which have an upstream domestic or no value chain path, and the third element ($\hat{v_C}\big [(I-A)^{-1}-(I-A_D)^{-1}\big ]\vec {e_i} \otimes \vec {e_i}^{T}{\hat{x}}^{-1}\big [(I-A)^{-1}-(I-A_D)^{-1}\big ]{\hat{f}}$) represents combinations of global downstream and upstream paths (simple-simple, simple-complex, complex-simple, complex-complex). All of these elements meet our definition of a complex global value chain because the value in all cases crosses borders for production at least twice.

##### Definition 9

No value chain tree $\tau _{i}^{NVC}$

$$\begin{aligned} \tau _{i}^{NVC}=&\, \hat{v_C}\vec {e_i} \otimes \vec {e_i}^{T}{\hat{x}}^{-1}{\hat{f}}. \end{aligned}$$

A no value chain tree represents that part of the output of each country-sector which is not part of a value chain according to Definition 6. In Fig. 1, a no value chain path is represented by the orange colour only (any other linkage represents a value chain path). Solely the share of value added produced in the respective country-sector in focus (no downstream stages) and also completed for final consumption (no upstream stages) in the same production phase satisfies this criterion. Since the I–O method distinguishes between a product used as an intermediate product within the same sector^24^ and the product manufactured for final consumption, the use of this definition as no value chain does not depend on the level of detail of I–O data disaggregation. The cyclical effect of the production of intermediate goods within the same country-sector is already included in the domestic value chain tree and, after taking into account all of the defined value chain paths (domestic, simple and complex global value chain paths), a value share remains without a value chain path and with a simple representation as the value added of the country-sector which is also directly consumed. This represents a value that has no path in terms of transactions that represent the fragmentation of production.

This concludes the value chain tree decomposition, which can be written as:

3.16 $$\begin{aligned}&\tau _{i}=\tau _{i}^{DVC}+\tau _{i}^{GVC}+\tau _{i}^{NVC}, \end{aligned}$$

3.17 $$\begin{aligned}&\tau _{i}^{GVC}=\tau _{i}^{SGVC}+\tau _{i}^{CGVC}. \end{aligned}$$

#### The value chain participation rates

In Sect. 3.1, we showed that a set of value chain tree matrices $\tau _i$ represents all possible value chain paths of the output of each country-sector and that the summation along all shares of total output assigned to all such unique value chain paths yields a unity for each value chain tree (Eq. 3.14). Namely, we presented a unique disaggregation of the output of each country-sector along all of its value chain paths. In the same way, the summation along the two disaggregating dimensions of our decomposed set of matrices (global, domestic and no value chain tree matrices) captures the overall share of the total output of each country-sector *i* that meets the criteria by which the value chain paths were decomposed by including either only domestic value chain paths, only global value chain paths, or only values that have no value chain paths at all. In other words, the summation of the disaggregated value chain matrices along any origin and end stage represents the share of output of each country-sector that has either a domestic, a global or a no value chain.

##### Definition 10

Domestic value chain share *DVCs*

$$DVCs \in \mathrm{I\!R}^n$$; $$DVCs_i = \sum _{j=1}^n \sum _{k=1}^n t_{ijk}^{DVC}$$; $$DVCs= \begin{bmatrix} {\mathbf {1}}^T \tau _{1}^{DVC} {\mathbf {1}} \\ {\mathbf {1}}^T \tau _{2}^{DVC} {\mathbf {1}} \\ \vdots \\ {\mathbf {1}}^T \tau _{n}^{DVC} {\mathbf {1}} \end{bmatrix}.$$

Domestic value chain share represents the share of each country-sector’s output that has a domestic value chain path.

##### Definition 11

Global value chain share *GVCs*

$$GVCs \in \mathrm{I\!R}^n$$; $$GVCs_i = \sum _{j=1}^n \sum _{k=1}^n t_{ijk}^{GVC}$$; $$GVCs= \begin{bmatrix} {\mathbf {1}}^T \tau _{1}^{GVC} {\mathbf {1}} \\ {\mathbf {1}}^T \tau _{2}^{GVC} {\mathbf {1}} \\ \vdots \\ {\mathbf {1}}^T \tau _{n}^{GVC} {\mathbf {1}} \end{bmatrix}.$$

Global value chain share represents the share of each country-sector’s output that has a global value chain path.

##### Definition 11.1

Simple global value chain share *SGVCs*

$$SGVCs \in \mathrm{I\!R}^n$$; $$SGVCs_i = \sum _{j=1}^n \sum _{k=1}^n t_{ijk}^{SGVC}$$; $$SGVCs= \begin{bmatrix} {\mathbf {1}}^T \tau _{1}^{SGVC} {\mathbf {1}} \\ {\mathbf {1}}^T \tau _{2}^{SGVC} {\mathbf {1}} \\ \vdots \\ {\mathbf {1}}^T \tau _{n}^{SGVC} {\mathbf {1}} \end{bmatrix}.$$

Simple global value chain share represents the share of each country-sector’s output that has a simple global value chain path.

##### Definition 11.2

Complex global value chain share *CGVCs*

$$CGVCs \in \mathrm{I\!R}^n$$; $$CGVCs_i = \sum _{j=1}^n \sum _{k=1}^n t_{ijk}^{CGVC}$$; $$CGVCs= \begin{bmatrix} {\mathbf {1}}^T \tau _{1}^{CGVC} {\mathbf {1}} \\ {\mathbf {1}}^T \tau _{2}^{CGVC} {\mathbf {1}} \\ \vdots \\ {\mathbf {1}}^T \tau _{n}^{CGVC} {\mathbf {1}} \end{bmatrix}.$$

Complex global value chain share represents the share of each country-sector’s output that has a complex global value chain path.

##### Definition 12

No value chain share *NVCs*

$$NVCs \in \mathrm{I\!R}^n$$; $$NVCs_i = \sum _{j=1}^n \sum _{k=1}^n t_{ijk}^{NVC}$$; $$NVCs= \begin{bmatrix} {\mathbf {1}}^T \tau _{1}^{NVC} {\mathbf {1}} \\ {\mathbf {1}}^T \tau _{2}^{NVC} {\mathbf {1}} \\ \vdots \\ {\mathbf {1}}^T \tau _{n}^{NVC} {\mathbf {1}} \end{bmatrix}.$$

A no value chain share represents the share of each country-sector’s output that has a no value chain path.

3.18 $$\begin{aligned}&DVCs_i+GVCs_i+NVCs_i=\sum _{j=1}^n \sum _{k=1}^n \bigg ( t_{ijk}^{DVC}+t_{ijk}^{GVC}+t_{ijk}^{NVC} \bigg )=\sum _{j=1}^n \sum _{k=1}^n t_{ijk}=1, \end{aligned}$$

3.19 $$\begin{aligned}&SGVCs_i+CGVCs_i=\sum _{j=1}^n \sum _{k=1}^n \bigg ( t_{ijk}^{SGVC}+t_{ijk}^{CGVC} \bigg )=\sum _{j=1}^n \sum _{k=1}^n t_{ijk}^{GVC}=GVCs_i. \end{aligned}$$

With this, we conclude our disaggregation of each country-sector’s total output with respect to its specific value chain integration based on production-sharing linkages. We can summarise our decomposition in the simple vector form:

3.20 $$\begin{aligned}&DVCs+GVCs+NVCs={\mathbf {1}}, \end{aligned}$$

3.21 $$\begin{aligned}&GVCs=SGVCs+CGVCs. \end{aligned}$$

#### Decomposition of the transaction to the final consumer

Since all value chain paths within production are covered and decomposed, we still have one last transaction to the consumer to complete the value chain path from production to consumption. We can decompose the final transaction to the consumer upon the criterion of whether it is a transaction to domestic consumers or a cross-border transaction (export of the final product for consumption). Domestic consumption here refers to the country-sector in which the last stage of production took place and not the country-sector whose value chain we are analysing. Each country-sector has a unique value chain and a specific structure of value chain paths. The completion of each value chain path by a transaction to the consumer can be achieved by an additional cross-border transaction of exporting the final product or consumption in the country where the product was finalised. Such a further decomposition of the value chain paths allows a more detailed analysis of the value chains.

The I–O data include information on the transaction to final consumers within matrix *F*, which can be decomposed into its cross-border and domestic flows to final consumers ($F=F_{CB} + F_{D}$) due to its block vector structure. We construct a matrix of all cross-border final consumption flows and a matrix of all domestic consumption flows:

3.22 $$\begin{aligned} {\hat{f}}={\hat{f}}_D+{\hat{f}}_{CB}. \end{aligned}$$

Every value chain path within production can thus be further decomposed with an additional criterion of a transaction to final consumers. Each set of disaggregated value chain matrices, defined by Eqs. 3.16 and 3.17, can be separated on two matrices, one covering all of the production paths that end in domestic final consumption (no export - $\tau _i^{NE}$) and the other all of the production value chain paths that end with exporting for final consumption ($\tau _i^{E}$).

3.23 $$\begin{aligned} \tau _i=\hat{v_C}(I-A)^{-1}\vec {e_i} \otimes \vec {e_i}^{T}{\hat{x}}^{-1}(I-A)^{-1}{\hat{f}}_D+\hat{v_C}(I-A)^{-1} \vec {e_i} \otimes \vec {e_i}^{T}{\hat{x}}^{-1}(I-A)^{-1}{\hat{f}}_{CB}=\tau _i^{NE}+\tau _i^E \end{aligned}$$

Due to their simple additive properties of operation, all of the decomposed value chain tree matrices are similarly decomposed to ones with exporting or with no exporting as the final transaction.

3.24 $$\begin{aligned}&\tau _i=\tau _i^{NE}+\tau _i^E=\tau _i^{GVC-NE}+\tau _i^{DVC-NE} +\tau _i^{NVC-NE}+\tau _i^{GVC-E}+\tau _i^{DVC-E}+\tau _i^{NVC-E} \end{aligned}$$

3.25 $$\begin{aligned}&\tau _i^{GVC}=\tau _i^{GVC-NE}+\tau _i^{GVC-E}= \tau _i^{SGVC-NE}+\tau _i^{CGVC-NE}+\tau _i^{SGVC-E}+\tau _i^{CGVC-E} \end{aligned}$$

The value shares that are part of each value chain path are thus further decomposed, as explained in Sect. 3.2.4. The final decomposition of the output is thus a decomposition along each value chain, as defined by criteria that simultaneously take account of transactions related to the production fragmentation (different value chains) and the final transaction to the consumer. A share of value that has either a domestic, global or no value chain has as its final transaction to the consumer either an export or a no export transaction, which provides a detailed decomposition of the participation shares that can be used to construct different composite indices suitable for different research questions.

3.26 $$\begin{aligned}&DVCs^{NE}+GVCs^{NE}+NVCs^{NE}+DVCs^{E}+GVCs^{E}+NVCs^{E}={\mathbf {1}} \end{aligned}$$

3.27 $$\begin{aligned}&GVCs=SGVCs^{NE}+CGVCs^{NE}+SGVCs^{E}+CGVCs^{E} \end{aligned}$$

## Results and discussion

The proposed measures broaden the scope for empirical application and static analysis of international production and trade. The contribution of our approach entails the simultaneous insight into domestic and global value chains, which allows the study of their interaction and structural changes in economies. All elements of the new typology may vary over time, from country to country and sector to sector and are relevant research topics. The derived participation shares are also simple fragmentation measures, and each smallest unit of analysis (country-sector) is represented by a single measure (scalar share) that covers the extent of overall value chain fragmentation, as opposed to separate downstream and upstream indicators.

**Fig. 2:**
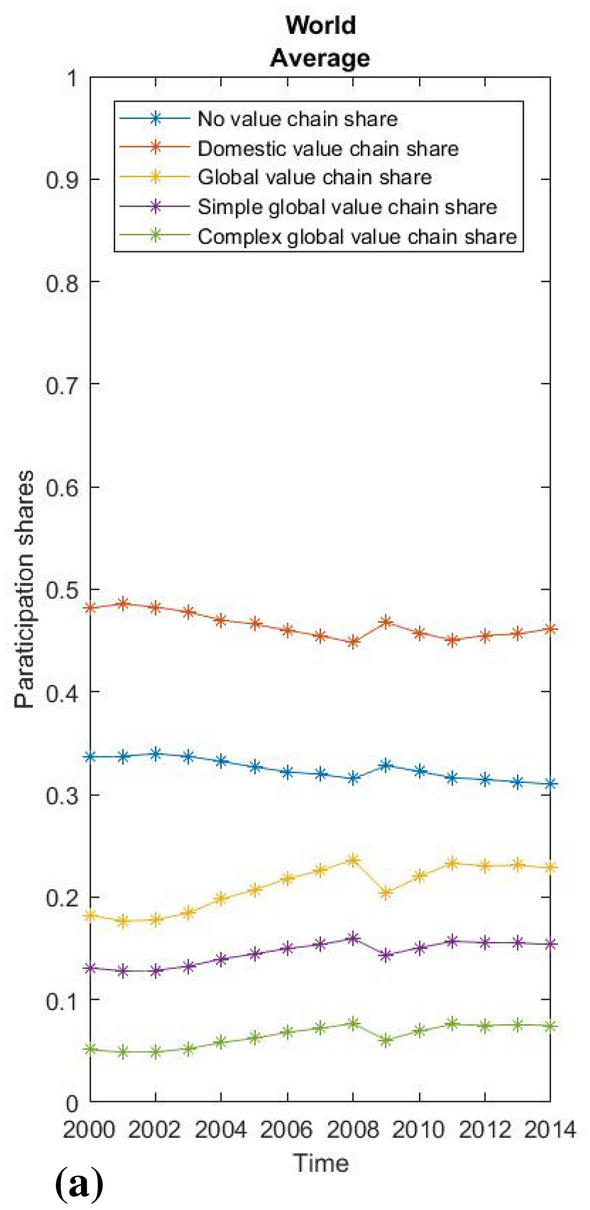
World average participation rates Source: WIOD, 2016; own calculations.

**Fig. 3:**
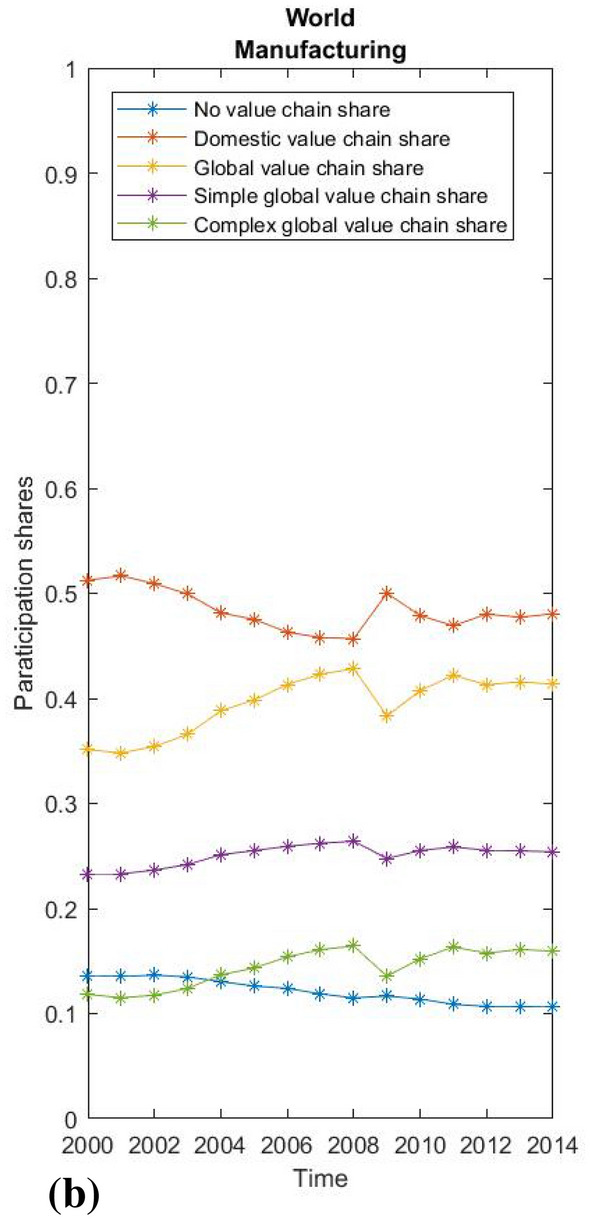
World average of manufacturing. Source: WIOD, 2016; own calculations

**Fig. 4:**
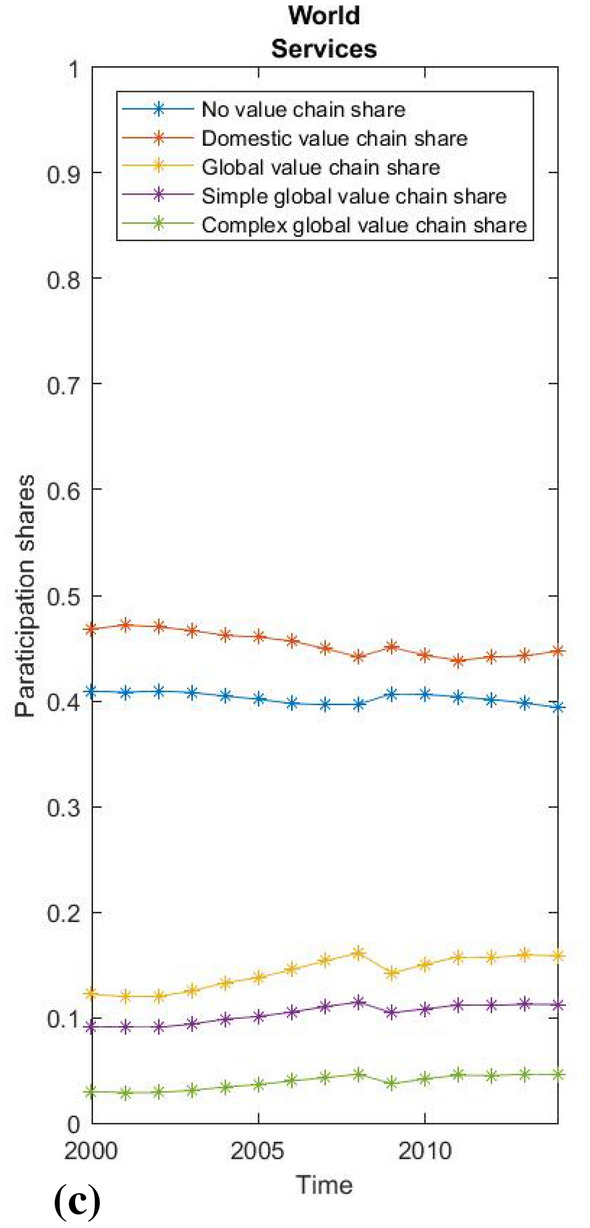
World average of services. Source: WIOD 2016; own calculations

Due to the limitations of the paper and its chiefly methodological focus, we present only some very basic empirical results. First, we show the global averages of value chain participation rates based on WIOD 2016 data and the global average participation rates for the manufacturing and service sectors separately (Figs. 2, 3 and 4). Using our methodological approach, we observe that the global average GVC share of world output consistently exceeds 20%, reached almost 24% at its peak before the global recession, and then stagnated slightly below this level until 2014 (Fig. 2). This suggests that the most recent estimates of GVCs’ share of production between 10 and 15% (Dollar 2017, p. 2; Li et al. 2019, p. 12) may be undervalued. As expected, the manufacturing sector is globally integrated to an above-average extent, with the share in the global value chain rising from 35 to over 40% before the crisis and then stagnating around this level after a brief recovery. The share of the complex global value chain shows the highest relative growth, while the average increase in global value chain integration exceeds the decline in domestic value chain integration. Interestingly, the decline in global integration in times of crisis had almost no impact on that part of the economy without value chain fragmentation, while domestic fragmentation increased almost in proportion to the decline in global integration. Hence, the crisis did not lead to a general decline in the fragmentation of production, but only to a decrease in its global character. For services, in contrast, less than 15% of total output has a global value chain path, although services show some increase in global integration, mainly due to decreasing domestic integration (which may be attributed to the globalisation of business services), while that part of the economy without a value chain appears relatively stable. For this reason, vulnerability to external financial shocks was much less pronounced in services during the crisis.

**Fig. 5:**
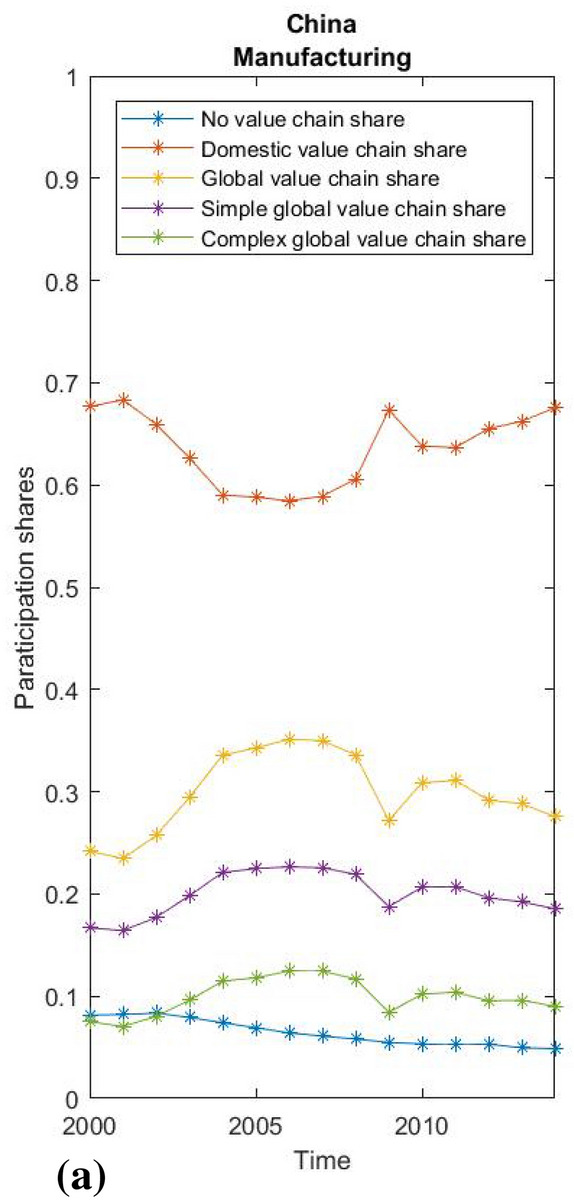
China manufacturing participation rates. Source: WIOD, 2016; own calculations

**Fig. 6:**
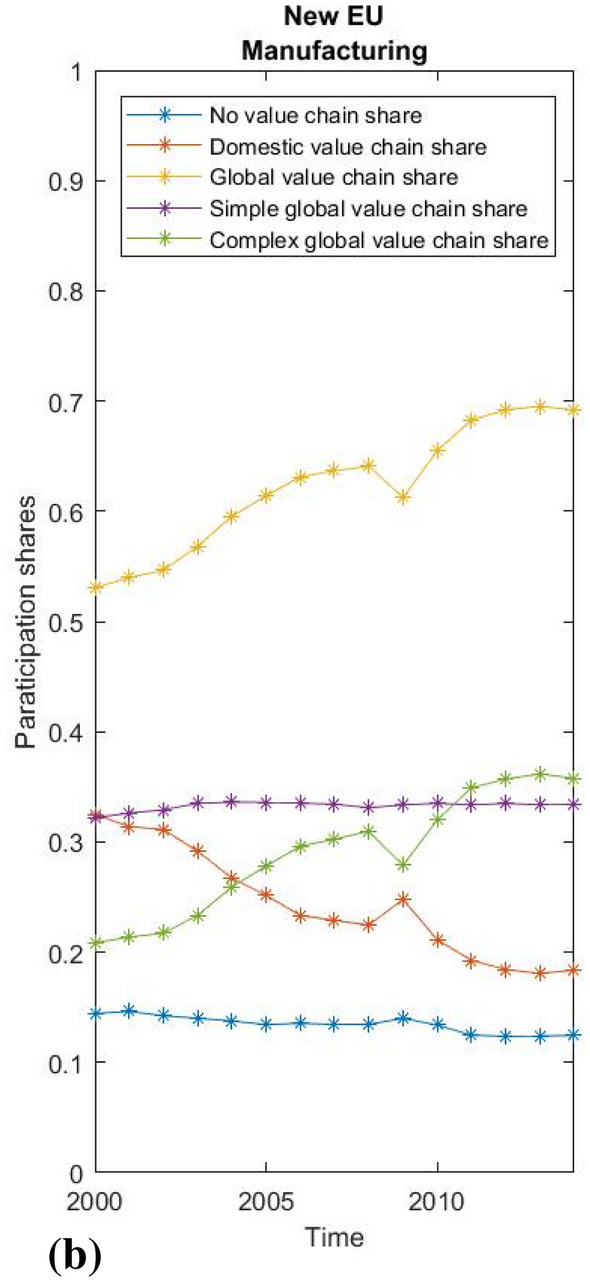
New EU countries manufacturing. Source: WIOD, 2016; own calculations

**Fig. 7:**
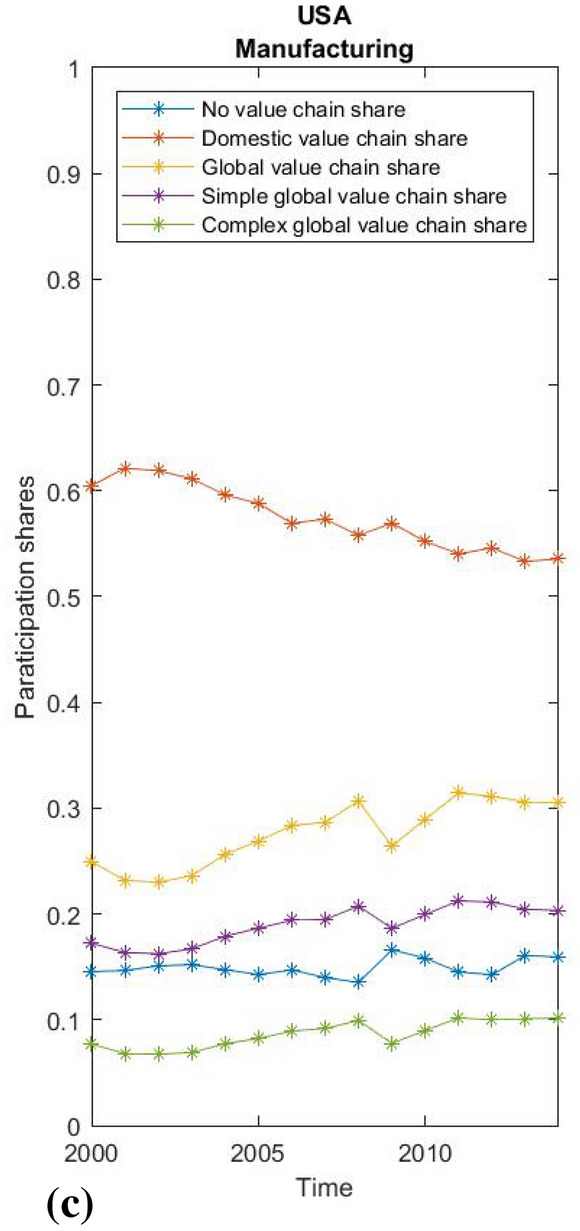
USA manufacturing participation rates. creditSource: WIOD 2016; own calculations

As the data for the world average conceal large differences between countries, we also show the value chain participation shares of manufacturing for China, the USA and the average of the economically most integrated new EU members—3 Baltic and 4 Visegrad countries (Figs. 5, 6 and 7), which reveal structural differences and diverse patterns of development in global and domestic integration. China has on average a high share of domestic production integration (around 65%) and is one of the few economies where the share of domestic integration grew by almost 10 percentage points between 2004 and 2014. In the United States, the picture is reversed, while the already lower average share of domestic integration is steadily shrinking. A completely different pattern is seen in the Baltic and Visegrad European countries, which became EU members in the new millennium. On average, these countries’ integration into global value chains in the manufacturing industries rose from an already high 53 to 69% during the observed period. At the same time, there was a huge relative decline in the already below-average share of domestic fragmentation from 32 to 18%. Interestingly, almost all of the growth in the global value chain share in Central and Eastern EU countries was due to the increase in complex global value chain linkages, while simple global value chain linkages remain relatively stable.

Finally, we use the fact that we have created uniform participation rates by performing a simple between-effects regression to test the relationship between the level of domestic and global fragmentation and economic growth measured by GDP per capita. Since short-term productivity fluctuations can hardly be explained by an economic structure expressed in value chain shares, we use a cross-sectional approach to test the long-term effects of different levels of domestic or global fragmentation on economic growth. Our observations relate to the 43 countries included in the WIOD 2016 data, and the variables are their average annual growth, the average DVC and GVC shares, with the average logarithm of GDP per capita as a control for convergence, the average logarithm of the annual population as a control for the size of the country, and the EU control dummy for potential EU specifics. The main regression equation with between effects is derived in the usual way out of a general panel data model:

$$y_{it}=\alpha _i + logGDP_{it}\beta _1+DVC_{it}\beta _2+GVC_{it}\beta _3+\epsilon _{it},$$

$$\overline{y_i}=\alpha +\overline{logGDP_i}\beta _1+\overline{DVC_i}\beta _2+\overline{GVC_i}\beta _3+ (\alpha _i-\alpha +\overline{\epsilon _{i}}).$$

To ensure a consistent estimator, $\alpha _i$ must be random effects. $y_{it}$ is yearly growth of GDP per capita, $logGDP_{it}$ is a logarithm of GDP per capita, while $DVC_{it}$ and $GVC_{it}$ represent shares of domestic and global value chains as calculated by the proposed methodology. The number of countries is 43 and number of time units is 15 (from 2000-2014), making a total of 645 observations in the panel.

The regression results are shown in Table 1. The logarithm of GDP per capita is a significant variable and negatively related to growth. The result simply reflects the fact that higher GDP implies less potential for higher growth rates, as implied by the convergence literature. Taking this into account, both the DVC share and the GVC share are highly significant variables that have a positive effect on growth rates. Therefore, both domestic and global integration can have a significant impact on economic growth. The same result applies after the introduction of additional controls on country size and EU specifics. Due to the principally methodological orientation of the article, we refrain from a detailed interpretation of the regression results. Yet, it should be noted that it is difficult to separate cause and effect while applying econometric analyses—a country in recession for external reasons could experience a decline in global and domestic production fragmentation due to those same external reasons. In any case, there is a correlation between economic growth and the degree of production fragmentation, whether it is domestic or global. A country that experiences an overall decrease in production fragmentation (domestic fragmentation declines faster than global increases), regardless of an increase in global production integration, might experience a negative impact on economic growth compared to similarly developed countries, in line with our findings.^25^ An increase only in participation in global value chains therefore does not necessarily enhance the growth due to various forms of integration^26^ with different effects on domestic integration, which is also an important factor in determining economic growth. Further studies are needed to examine the relationship between domestic and global fragmentation and diverse patterns of structural integration that could also help in assessing the impact of unpredictable circumstances (e.g. COVID 19) on individual countries, regions or sectors.

**Table 1:** Regression results Source: WIOD, 2016; WB; own calculations

	(1)	(2)	(3)
	Yearly growth	Yearly growth	Yearly growth
logGDP	− 0.013***	− 0.013***	− 0.013***
	(0.00)	(0.00)	(0.00)
DVC share	0.169***	0.181***	0.183***
	(0.03)	(0.04)	(0.04)
GVC share	0.163***	0.160***	0.162***
	(0.03)	(0.03)	(0.03)
logPOP		− 0.001	− 0.001
		(0.00)	(0.00)
EU			− 0.003
			(0.00)
Constant	0.036	0.049	0.055
	(0.03)	(0.03)	(0.04)
$R^2$	0.819	0.821	0.824
*F*	57.126	42.314	33.806

* $$p<0.05$$, ** $$p<0.01$$, *** $$p<0.001$$

## Conclusion

We have proposed a new methodology for measuring the participation shares of different types of value chains in the international input–output framework. We addressed the lack of a consistent unitary measure of value chain integration on the country-sector level by proposing a new concept of the value chain tree for each country-sector, covering all value chain paths from value creation (downstream linkages) through a single country-sector to final consumption (upstream linkages) simultaneously. By capturing the structure of all value chains in a series of value chain tree matrices, we add a new mathematical object that serves as a basis for deriving the proposed new indicator of value chain participation, which we contribute to the existing collection of indicators.

This methodology allows us to introduce an extended typology of value chains by distinguishing and disaggregating all production activity into the following types: no value chain, domestic value chain, and global value chain—further differentiated into simple and complex global value chains. The most important new conceptual subdivision in the extended typology relates to the subdivision of the existing ’domestic component’ into a no value chain and a domestic value chain. This subdivision, which is only possible with the proposed methodology, provides a better representation of domestic production interdependencies and permits comparative analyses of the simultaneous development of domestic and foreign production interdependencies, thereby enabling aggregated analyses of domestic and global production fragmentation and its interrelated development as influenced by outsourcing or offshoring. Another big change introduced by the new typology is its fundamental production-related character: all distinctions between different types of value chains are made only with regard to (potential) production fragmentation, with a separate examination of the transaction to the final consumer—which may or may not be cross-border. This affirms the concept of value chain as related primarily to the fragmentation of production, while the *post festum* differentiation is also derived based on the last transaction to the final consumer.

The proposed methodology and typology of value chains provides researchers with new opportunities to conduct future research on different levels of disaggregation, be it comparative geographical analysis (e.g. comparing the evolution of value chain measures between two countries or between groups of countries) or observing the evolution of value chains in different sectoral disaggregations. The preliminary illustration of the new methodology, which attempts to link both domestic and global production fragmentation with long-term growth rates, shows a positive correlation between both global and domestic production fragmentation with economic growth. This result may indicate that it is the general complexity of the division of labour, reflected in the general fragmentation of production, that is chiefly correlated with growth, irrespective of its global or domestic nature. Accordingly, the proposed measure and the new typology of value chains, in particular the novel conceptualisation of domestic value chain fragmentation, could bring to light important information that has been concealed in the existing typology, which conceptualises the domestic component only as a negation of the global value chain and thus did not allow research with explicit questions concerning domestic integration. The complex development of globalisation in recent decades and the shifts of late towards the localisation and regionalisation of economic integration caused by political, economic and external factors make this new approach increasingly relevant. The proposed measure, particularly in conjunction with data from other sources, could further deepen the theoretical discussion and empirical investigations.

In conclusion, we believe that our new methodological approach and the new extended typology of value chains associated with it provide fertile grounds for obtaining deeper insights into different types of value chains as well as a broader set of tools of use for various extensions of research.

## Data Availability

The datasets analysed during the current study are available at http://www.wiod.org.

## Appendix A: Notations

$n_S\in \mathrm{I\!N}$ Number of sectors.

$n_C\in \mathrm{I\!N}$ Number of countries.

$n\in \mathrm{I\!N}$; $n=n_S*n_C$ Number of country-sectors.

${\mathbf {1}}\in \mathrm{I\!R}^n$ Vector of ones.

$\vec {1}\in \mathrm{I\!R}^{n_C}$ vector of ones.

$\vec {e_i} \in \mathrm{I\!R}^n$; ${e_i}_j=\delta _{ij}$ Standard orthonormal basis of $\mathrm{I\!R}^n$.

$$I \in \mathrm{I\!R}^{n\times n}$$ Identity matrix.

$x \in \mathrm{I\!R}^n$ Total output vector.

${\hat{x}} \in \mathrm{I\!R}^{n\times n}$; ${\hat{x}}=diag(x)$ Total output matrix.

$C \in \mathrm{I\!R}^{n\times n}$ Intermediate consumption matrix.

$F\in \mathrm{I\!R}^{n\times n_C}$ Final consumption matrix on the country level.^27^

$f \in \mathrm{I\!R}^n$; $f=F\vec {1}$ Total final consumption vector.

${\hat{f}}\in \mathrm{I\!R}^{n\times n}$; ${\hat{f}}=diag(f)$ Total final consumption matrix.

$A \in \mathrm{I\!R}^{n\times n}$; $A =C{\hat{x}}^{-1}$ Leontief technical coefficient matrix.

$G \in \mathrm{I\!R}^{n\times n}$; $G ={\hat{x}}^{-1}C$ Ghosh technical coefficient matrix.

$v \in \mathrm{I\!R}^n$; $v^T= x^T-{\mathbf {1}}^TC={\mathbf {1}}({\hat{x}}-A{\hat{x}})={\mathbf {1}}^T(I-A){\hat{x}}$ Vector of total value added.

${\hat{v}}\in \mathrm{I\!R}^{n\times n}$; ${\hat{v}}=diag(v)$ Total value-added matrix.

$v_C \in \mathrm{I\!R}^n$; $v_C^T= v^T{\hat{x}}^{-1}={\mathbf {1}}^T(I-A)$ Vector of value-added coefficients – value-added share in total output.

${\hat{v}}_C\in \mathrm{I\!R}^{n\times n}$; ${\hat{v}}_C=diag(v_C)$ Value-added coefficients matrix.

*C*, *A* and *G* have a block-matrix structure $\mathrm{I\!R}^{(n_S\times n_S)\times (n_C\times n_C) }$, while *F* has a block vector structure $\mathrm{I\!R}^{n_S \times (n_C\times n_C)}$. Diagonal block elements with respect to countries represent domestic intermediate transfers and domestic consumption and off diagonal block elements represent transactions that cross a border either for intermediate use or final consumption.

$C=C_{CB} + C_{D}$ $A=A_{CB} + A_{D}$ $G=G_{CB} + G_{D}$ $F=F_{CB} + F_{D}$ $f_{CB} \in \mathrm{I\!R}^n$; $f_{CB}=F_{CB}\vec {1}$ Total final consumption by exporting.

$f_{D} \in \mathrm{I\!R}^n$; $f_{D}=F_{D}\vec {1}$ Total final consumption by domestic transactions.

${\hat{f}}_{CB}\in \mathrm{I\!R}^{n\times n}$; ${\hat{f}}_{CB}=diag(f_{CB})$ Total final consumption by exporting matrix.

$${\hat{f}}_{D}\in \mathrm{I\!R}^{n\times n}$$;

${\hat{f}}_{D}=diag(f_{D})$ Total final consumption by domestic transactions matrix.

## Appendix B: $\tau _i$ decomposition

$$\begin{aligned} \tau _i &= \hat{v_C}(I-A)^{-1}\vec {e_i} \otimes \vec {e_i}^{T}{\hat{x}}^{-1}(I-A)^{-1}{\hat{f}} \\ &=\hat{v_C}\bigg [I+A_D(I-A_D)^{-1}+(I-A_D)^{-1}A_{CB}(I-A_D)^{-1} \\ &\quad+(I-A)^{-1}-(I-A_D)^{-1}-(I-A_D)^{-1}A_{CB}(I-A_D)^{-1}\bigg ]\vec {e_i} \\ &\quad \otimes \vec {e_i}^{T}{\hat{x}}^{-1}\bigg [I+A_D(I-A_D)^{-1} +(I-A_D)^{-1}A_{CB}(I-A_D)^{-1} \\ &\quad+(I-A)^{-1}-(I-A_D)^{-1}-(I-A_D)^{-1}A_{CB}(I-A_D)^{-1}\bigg ]{\hat{f}} \\ &=\hat{v_C}\vec {e_i} \otimes \vec {e_i}^{T}{\hat{x}}^{-1}{\hat{f}}+\hat{v_C}A_D(I-A_D)^{-1}\vec {e_i} \otimes \vec {e_i}^{T}{\hat{x}}^{-1}{\hat{f}}+ \hat{v_C}\vec {e_i} \otimes \vec {e_i}^{T}{\hat{x}}^{-1}A_D(I-A_D)^{-1}{\hat{f}} \\ &\quad +\hat{v_C}A_D(I-A_D)^{-1}\vec {e_i} \otimes \vec {e_i}^{T}{\hat{x}}^{-1}A_D(I-A_D)^{-1}{\hat{f}}+\hat{v_C}\vec {e_i} \otimes \vec {e_i}^{T}{\hat{x}}^{-1}(I-A_D)^{-1}A_{CB}(I-A_D)^{-1}{\hat{f}} \\ &\quad+ \hat{v_C}A_D(I-A_D)^{-1}\vec {e_i} \otimes \vec {e_i}^{T}{\hat{x}}^{-1}(I-A_D)^{-1}A_{CB}(I-A_D)^{-1}{\hat{f}} +\hat{v_C}(I-A_D)^{-1}A_{CB}(I-A_D)^{-1}\vec {e_i} \otimes \vec {e_i}^{T}{\hat{x}}^{-1}{\hat{f}} \\ &\quad+\hat{v_C}(I-A_D)^{-1}A_{CB}(I-A_D)^{-1}\vec {e_i} \otimes \vec {e_i}^{T}{\hat{x}}^{-1}A_D(I-A_D)^{-1}{\hat{f}} \\ &\quad+\hat{v_C}(I-A_D)^{-1}A_{CB}(I-A_D)^{-1}\vec {e_i} \otimes \vec {e_i}^{T}{\hat{x}}^{-1}(I-A_D)^{-1}A_{CB}(I-A_D)^{-1}{\hat{f}} \\ &\quad+\hat{v_C}\vec {e_i} \otimes \vec {e_i}^{T}{\hat{x}}^{-1}\big ((I-A)^{-1}-(I-A_D)^{-1}-(I-A_D)^{-1} A_{CB}(I-A_D)^{-1}\big ){\hat{f}} \\ &\quad+\hat{v_C}A_D(I-A_D)^{-1}\vec {e_i} \otimes \vec {e_i}^{T}{\hat{x}}^{-1}\big ((I-A)^{-1}-(I-A_D)^{-1}-(I-A_D)^{-1} A_{CB}(I-A_D)^{-1}\big ){\hat{f}} \\ &\quad+\hat{v_C}(I-A_D)^{-1}A_{CB}(I-A_D)^{-1}\vec {e_i} \otimes \vec {e_i}^{T}{\hat{x}}^{-1}\big ((I-A)^{-1}-(I-A_D)^{-1}-(I-A_D)^{-1} A_{CB}(I-A_D)^{-1}\big ){\hat{f}} \\ &\quad+\hat{v_C}\big ((I-A)^{-1}-(I-A_D)^{-1}-(I-A_D)^{-1}A_{CB}(I-A_D)^{-1} \big )\vec {e_i} \otimes \vec {e_i}^{T}{\hat{x}}^{-1}{\hat{f}} \\ &\quad+\hat{v_C}\big ((I-A)^{-1}-(I-A_D)^{-1}-(I-A_D)^{-1}A_{CB}(I-A_D)^{-1} \big )\vec {e_i} \otimes \vec {e_i}^{T}{\hat{x}}^{-1}A_D(I-A_D)^{-1}{\hat{f}} \\ &\quad+\hat{v_C}\big ((I-A)^{-1}-(I-A_D)^{-1}-(I-A_D)^{-1}A_{CB}(I-A_D)^{-1} \big )\vec {e_i} \otimes \vec {e_i}^{T}{\hat{x}}^{-1}(I-A_D)^{-1}A_{CB}(I-A_D)^{-1}{\hat{f}} \\ &\quad+\hat{v_C}\big ((I-A)^{-1}-(I-A_D)^{-1}-(I-A_D)^{-1}A_{CB}(I-A_D)^{-1} \big )\vec {e_i} \\ &\quad\otimes \vec {e_i}^{T}{\hat{x}}^{-1}\big ((I-A)^{-1}-(I-A_D)^{-1}-(I-A_D)^{-1} A_{CB}(I-A_D)^{-1}\big ) {\hat{f}} \\ &=\hat{v_C}\vec {e_i} \otimes \vec {e_i}^{T}{\hat{x}}^{-1}{\hat{f}}+\hat{v_C}A_D(I-A_D)^{-1}\vec {e_i} \otimes \vec {e_i}^{T}{\hat{x}}^{-1}{\hat{f}}+ \hat{v_C}\vec {e_i} \otimes \vec {e_i}^{T}{\hat{x}}^{-1}A_D(I-A_D)^{-1}{\hat{f}} \\ &\quad+\hat{v_C}A_D(I-A_D)^{-1}\vec {e_i} \otimes \vec {e_i}^{T}{\hat{x}}^{-1}A_D(I-A_D)^{-1}{\hat{f}}+\hat{v_C}(I-A_D)^{-1} \vec {e_i} \otimes \vec {e_i}^{T}{\hat{x}}^{-1}\big ((I-A)^{-1}-(I-A_D)^{-1}\big ){\hat{f}} \\ &\quad+\hat{v_C}\big ((I-A)^{-1}-(I-A_D)^{-1}\big )\vec {e_i} \otimes \vec {e_i}^{T}{\hat{x}}^{-1}(I-A_D)^{-1}{\hat{f}} \\&\quad +\hat{v_C}\big ((I-A)^{-1}-(I-A_D)^{-1}\big )\vec {e_i} \otimes \vec {e_i}^{T}{\hat{x}}^{-1}\big ((I-A)^{-1}-(I-A_D)^{-1}\big ){\hat{f}} \\ &=\tau _{i}^{\text{NVC}}+\tau _{i}^{\text{DVC}}+\tau _{i}^{\text{GVC}}=\tau _{i}. \end{aligned}$$

$$\begin{aligned} \tau _{i}^{\text{GVC}}= & {} \hat{v_C}(I-A_D)^{-1}\vec {e_i} \otimes \vec {e_i}^{T}{\hat{x}}^{-1}\big ((I-A)^{-1}-(I-A_D)^{-1}\big ){\hat{f}} \\&+\hat{v_C}\big ((I-A)^{-1}-(I-A_D)^{-1}\big )\vec {e_i} \otimes \vec {e_i}^{T}{\hat{x}}^{-1}(I-A_D)^{-1}{\hat{f}} \\&+ \hat{v_C}\big ((I-A)^{-1}-(I-A_D)^{-1}\big )\vec {e_i} \otimes \vec {e_i}^{T}{\hat{x}}^{-1}\big ((I-A)^{-1}-(I-A_D)^{-1}\big ){\hat{f}} \\&=\hat{v_C}(I-A_D)^{-1}\vec {e_i} \otimes \vec {e_i}^{T}{\hat{x}}^{-1}(I-A_D)^{-1}A_{CB}(I-A_D)^{-1}{\hat{f}} \\&+\hat{v_C}(I-A_D)^{-1}A_{CB}(I-A_D)^{-1}\vec {e_i} \otimes \vec {e_i}^{T}{\hat{x}}^{-1}(I-A_D)^{-1}{\hat{f}} \\&+\hat{v_C}(I-A_D)^{-1}\vec {e_i} \otimes \vec {e_i}^{T}{\hat{x}}^{-1}\big [(I-A)^{-1}-(I-A_D)^{-1}-(I-A_D)^{-1} A_{CB}(I-A_D)^{-1}\big ]{\hat{f}} \\&+\hat{v_C}\big [(I-A)^{-1}-(I-A_D)^{-1}-(I-A_D)^{-1}A_{CB}(I-A_D)^{-1} \big ]\vec {e_i} \otimes \vec {e_i}^{T}{\hat{x}}^{-1}(I-A_D)^{-1}{\hat{f}} \\&+\hat{v_C}\big [(I-A)^{-1}-(I-A_D)^{-1}\big ]\vec {e_i} \otimes \vec {e_i}^{T}{\hat{x}}^{-1}\big [(I-A)^{-1}-(I-A_D)^{-1}\big ]{\hat{f}} \\&=\tau _{i}^{\text{SGVC}}+\tau _{i}^{\text{CGVC}}=\tau _{i}^{\text{GVC}}. \end{aligned}$$

## Appendix C

We make a demonstration of the methodology on a simple 2 sector 2 countries numerical example.^28^ This simple case of international economy has following intermediate consumption matrix and final demand:

$$\begin{aligned} C=\begin{bmatrix} 2 &{} 1 &{} 1 &{} 2\\ 0.5 &{} 0.33 &{} 0.33 &{} 0.33\\ 0.5 &{} 0.5 &{} 0.167 &{} 0.33\\ 1&{} 1.5 &{} 2 &{}2 \end{bmatrix} \qquad f=\begin{bmatrix} 4 \\ 2 \\ 3 \\ 5 \end{bmatrix}. \end{aligned}$$

Total output is the sum of all the intermediate and final demand:

$$\begin{aligned} x=C{\mathbf {1}}+f\qquad x=\begin{bmatrix} 10\\ 3.5\\ 4.5\\ 11.5 \end{bmatrix}. \end{aligned}$$

Calculation of value added coefficients and Leontief technical coefficients:

$$\begin{aligned} v_C^T={\mathbf {1}}^T(I-A)=\begin{bmatrix} 0.6&0.0476&0.2222&0.5942\end{bmatrix}, \\ A=C{\hat{x}}^{-1}=\begin{bmatrix} 0.2 &{} 0.2857 &{} 0.2222 &{} 0.1739 \\ 0.05 &{} 0.09523 &{} 0.0740 &{} 0.0289 \\ 0.05 &{} 0.1428 &{} 0.0370 &{} 0.0289 \\ 0.1 &{} 0.4285 &{} 0.4444 &{} 0.1739 \end{bmatrix}, \\ A_{\text{Dom}}=\begin{bmatrix} 0.2 &{} 0.2857 &{} 0&{} 0 \\ 0.05 &{} 0.09523 &{} 0 &{} 0\\ 0 &{} 0 &{} 0.0370 &{} 0.0289 \\ 0 &{} 0 &{} 0.4444 &{} 0.1739 \end{bmatrix}\qquad A_{CB}=\begin{bmatrix} 0 &{} 0 &{} 0.2222 &{} 0.1739 \\ 0 &{} 0&{} 0.0740 &{} 0.0289 \\ 0.05 &{} 0.1428 &{} 0 &{} 0 \\ 0.1 &{} 0.4285 &{} 0 &{} 0 \end{bmatrix}. \end{aligned}$$

We continue with separate upstream and downstream decompositions, *W* and *Z*:

$$\begin{aligned} W= & {} {\hat{x}}^{-1}(I-A)^{-1}{\hat{f}}=\begin{bmatrix} 0.5461 &{} 0.1337 &{} 0.1555&{} 0.1645\\ 0.1044&{} 0.6788&{} 0.1224&{} 0.0942\\ 0.0820&{} 0.1047&{} 0.7391&{} 0.0740\\ 0.09126&{} 0.1433&{} 0.1913&{} 0.5740 \end{bmatrix},\\ Z= & {} {\hat{v}}_C(I-A)^{-1}=\begin{bmatrix} 0.8192 &{} 0.4013 &{} 0.3110 &{} 0.1974 \\ 0.0043 &{} 0.0565&{} 0.0068&{} 0.0031\\ 0.0205&{} 0.0523&{} 0.2463&{} 0.0148\\ 0.1559&{} 0.4896&{} 0.4357&{} 0.7845 \end{bmatrix}. \end{aligned}$$

Value chain tree matrices are calculated for each country-sector in the following manner:

$$\begin{aligned} \tau _i=Z\vec {e_i} \otimes \vec {e_i}^{T}W \\ \tau _1=\begin{bmatrix} 0.4474&{} 0.1095&{} 0.1274&{}0.1348\\ 0.0023&{} 0.0005&{} 0.0006&{} 0.0007\\ 0.0112&{} 0.0027&{} 0.0031&{} 0.0033\\ 0.0851&{} 0.0208&{} 0.0242&{} 0.0256 \end{bmatrix},\qquad \tau _2= \begin{bmatrix} 0.0419&{} 0.2724&{} 0.0491&{} 0.0378\\ 0.0059&{} 0.0383&{}0.0069&{} 0.0053\\ 0.0054&{}0.03556&{} 0.0064&{} 0.0049\\ 0.0511&{} 0.3324&{}0.0599&{} 0.0461 \end{bmatrix}, \\ \tau _3=\begin{bmatrix} 0.0255&{} 0.0325&{}0.2299&{} 0.0230\\ 0.0005&{} 0.0007&{} 0.0050&{} 0.0005\\ 0.0202&{} 0.0258&{} 0.1820&{} 0.0182\\ 0.0357&{} 0.0456&{}0.3220&{}0.0322 \end{bmatrix}, \qquad \tau _4= \begin{bmatrix} 0.0180&{} 0.0283&{} 0.0377&{} 0.1133\\ 0.0002&{} 0.0004&{} 0.0006&{}0.0018\\ 0.0013&{} 0.0021&{} 0.0028&{} 0.0084\\ 0.0716&{} 0.1124&{} 0.1501&{}0.4504 \end{bmatrix}. \end{aligned}$$

For each type of value chain (DVC, GVC, NVC,...) we have 4 matrices, each covering all the value chain paths of each country-sector (we have 4 in our example) that conform to our value chain criteria.

$$\begin{aligned} \tau ^{\text{DVC}}_1= & {} \begin{bmatrix} 0.1502&{} 0.0616&{} 0 &{}0\\ 0.0017&{} 0.0002&{} 0&{} 0\\ 0 &{}0 &{}0&{} 0\\ 0&{} 0 &{}0&{} 0 \end{bmatrix}\qquad \tau _2^{\text{DVC}}=\begin{bmatrix} 0.0194&{} 0.1556&{} 0 &{}0\\ 0.0043&{} 0.0073&{}0&{} 0\\ 0&{} 0 &{}0 &{}0\\ 0 &{}0&{} 0&{} 0 \end{bmatrix} \\ \tau ^{\text{DVC}}_3= & {} \begin{bmatrix} 0&{} 0&{} 0&{} 0\\ 0 &{}0&{} 0&{} 0\\ 0&{} 0&{} 0.0169&{} 0.0096\\ 0&{} 0&{} 0.2374&{} 0.0138 \end{bmatrix}\qquad \tau ^{\text{DVC}}_4= \begin{bmatrix} 0 &{}0&{} 0&{} 0\\ 0&{} 0&{} 0&{} 0\\ 0&{} 0&{} 0.0012&{} 0.0044\\ 0&{} 0&{} 0.1083&{} 0.1327 \end{bmatrix} \\ \tau ^{\text{GVC}}_1= & {} \begin{bmatrix} 0.0571&{} 0.0479&{} 0.1274&{}0.1348\\ 0.0006&{} 0.0003&{} 0.0006&{} 0.0007\\ 0.0112&{}0.0027&{} 0.0031&{} 0.0033\\ 0.0851&{} 0.0208&{}0.0242&{} 0.0256 \end{bmatrix} \qquad \tau ^{\text{GVC}}_2=\begin{bmatrix} 0.0224&{}0.1167&{} 0.0491&{} 0.0378\\ 0.0015&{}9 0.0038&{} 0.0069&{} 0.0053\\ 0.0054&{} 0.0355&{} 0.0064&{} 0.0049\\ 0.0511&{} 0.3324&{} 0.0599&{} 0.0461 \end{bmatrix}\\ \tau ^{\text{GVC}}_3= & {} \begin{bmatrix} 0.0255&{} 0.0325&{} 0.2299&{} 0.0230\\ 0.0005&{} 0.0007&{} 0.0050&{} 0.0005\\ 0.0202&{} 0.0258&{} 0.0170&{} 0.0085\\ 0.0357&{} 0.0456&{} 0.0846&{} 0.0183 \end{bmatrix} \qquad \tau ^{\text{GVC}}_4=\begin{bmatrix} 0.0180&{} 0.0283&{} 0.0377&{}0.1133\\ 0.0002&{} 0.0004&{} 0.0006&{} 0.0018\\ 0.0013&{} 0.0021&{} 0.0016&{}0.0040\\ 0.0716&{} 0.1124&{} 0.0417&{} 0.0592 \end{bmatrix}\\ \tau ^{\text{NVC}}_1= & {} \begin{bmatrix} 0.24 &{} 0 &{} 0 &{} 0\\ 0 &{} 0 &{} 0 &{} 0\\ 0 &{} 0 &{} 0 &{} 0\\ 0 &{} 0 &{} 0 &{} 0 \end{bmatrix}\qquad \tau ^{\text{NVC}}_1= \begin{bmatrix} 0 &{} 0 &{} 0 &{} 0\\ 0 &{} 0.0272 &{} 0 &{} 0\\ 0 &{} 0 &{} 0 &{} 0\\ 0 &{} 0 &{} 0 &{} 0 \end{bmatrix}\\ \tau ^{\text{NVC}}_3= & {} \begin{bmatrix} 0 &{} 0 &{} 0 &{} 0\\ 0 &{} 0 &{} 0 &{} 0\\ 0 &{} 0 &{} 0.1481 &{} 0\\ 0 &{} 0 &{} 0 &{} 0 \end{bmatrix}\qquad \tau ^{\text{NVC}}_4= \begin{bmatrix} 0 &{} 0 &{} 0 &{} 0\\ 0 &{} 0 &{} 0 &{} 0\\ 0 &{} 0 &{} 0 &{} 0\\ 0 &{} 0 &{} 0 &{} 0.2583 \end{bmatrix} \end{aligned}$$

The value chain participation shares are obtained by summation of all elements of the value chain tree matrices:

$$\begin{aligned} {\text{DVCs}}= & {} \begin{bmatrix} {\mathbf {1}}^T \tau _{1}^{\text{DVC}} {\mathbf {1}} \\ {\mathbf {1}}^T \tau _{2}^{\text{DVC}} {\mathbf {1}} \\ {\mathbf {1}}^T \tau _{3}^{\text{DVC}} {\mathbf {1}} \\ {\mathbf {1}}^T \tau _{4}^{\text{DVC}} {\mathbf {1}} \\ \end{bmatrix}=\begin{bmatrix} 0.2138\\ 0.1868\\ 0.2779\\ 0.2467\\ \end{bmatrix},\\ {\text{GVCs}}= & {} \begin{bmatrix} {\mathbf {1}}^T \tau _{1}^{\text{GVC}} {\mathbf {1}} \\ {\mathbf {1}}^T \tau _{2}^{\text{GVC}} {\mathbf {1}} \\ {\mathbf {1}}^T \tau _{3}^{\text{GVC}} {\mathbf {1}} \\ {\mathbf {1}}^T \tau _{4}^{\text{GVC}} {\mathbf {1}} \\ \end{bmatrix}=\begin{bmatrix} 0.5461\\ 0.7859\\ 0.5739\\ 0.4949 \end{bmatrix},\\ {\text{NVCs}}= & {} \begin{bmatrix} {\mathbf {1}}^T \tau _{1}^{\text{NVC}} {\mathbf {1}} \\ {\mathbf {1}}^T \tau _{2}^{\text{NVC}} {\mathbf {1}} \\ {\mathbf {1}}^T \tau _{3}^{NVC} {\mathbf {1}} \\ {\mathbf {1}}^T \tau _{4}^{\text{NVC}} {\mathbf {1}} \\ \end{bmatrix}=\begin{bmatrix} 0.24\\ 0.0272\\ 0.1481\\ 0.2583 \end{bmatrix}. \end{aligned}$$

## Footnotes

1 The term global commodity chain is a predecessor of global value chain.

2 Embracing a historical and macroeconomic approach to the analysis of the global division of labour, the world-systems approach examines the unequal patterns of exchange along global commodity chains as well as different structural patterns of the international integration of the core, periphery and semi-periphery (Arrighi and Drangel 1986).

3 Governance was conceived as either consumer-driven (apparel sector) or producer-driven (automotive sector). This approach was further extended by Ponte and Sturgeon (2013).

4 Porter’s (1985) concept of the intra-firm value chain is often used to discuss the specialisation of enterprises, and core competencies and business literature on multinational enterprises overlap with the global value chain framework.

5 Which was used to extend the producer-driven and consumer-driven governance typology of commodity chain research to a more general typology of value chain linkages, from transactions in a completely free market to a strict hierarchy (Gereffi et al. 2005).

6 In international economics, use of the input–output methodology grew in importance as researchers of various international incentives integrated nationally based input–output tables into harmonised global input–output tables. The most prominent are the World Input–Output Database (Timmer et al. 2015), the OECD’s Trade in Value Added and the EORA (Lenzen et al. 2013).

7 While all heterogeneous approaches to value chains focus on a development issue, the recent GVC approach has been adopted by international institutions to highlight the gains from liberalisation and industrial upgrading, while the world-systems approach critically examines unequal rewards along the value chain and different structural integration patterns that may cause the perpetuation of unequal development (Gereffi 2018; Taglioni and Winkler 2016).

8 Relative position indices can easily be derived from length measures as simple ratios.

9 Using a method similar to that used to calculate the average propagation length required for the analysis of the dynamic response to shocks, defined by Dietzenbacher and Romero (2016).

10 It is also obvious that a simple solution, such as using the average of existing upstream and downstream indicators, cannot be justified in theory. If, for example, a given country-sector’s share in the upstream global value chain is high (close to 100%) and its share in the downstream global value chain is relatively low (close to 0%), then the average share in the value chain would be around 50%, which is misleading because the value chain as a whole is almost entirely global (using the criterion that the value crosses a border at least once). As far as value chain paths are concerned, despite the small share of downstream global value chain paths, a high share (close to 100%) of the same paths continues in the upstream global value chain such that production as a whole has a very high global share (close to 100%), while the use of the average of the upstream and downstream indicators does not correspond to the definition of the global value chain.

11 For example, in a forthcoming article we explore the decomposition of value chains based on the criterion of the number of domestic transactions subject to meeting the usual global value chain criterion of having at least one production-sharing cross-border transaction. In this setting, we decompose the global value chain share into a GVC with no domestic cooperation, a GVC with simple domestic cooperation, and a GVC with complex domestic cooperation, offering information on the specific pattern of the EU periphery’s integration.

12 For example, the concept of integrated periphery was introduced to describe a specific type of integration in the case of the Slovak and Czech car industries, characterised by their proximity to consumer markets, cheaper labour force, the absence of positive spillover effects and lack of domestic linkages (Oldřich and Vladan 2019; Pavlínek 2018).

13 In our derivation, which is consistent with most existing international I–O data, the country-sector is the smallest object of analysis. When we refer to our methodology and derive it, the reference to the country-sector refers to the smallest object of analysis given by the level of detail of the I–O data set. If the I–O data sets were built on a more detailed structure at the enterprise level (greatly increasing the dimension), the proposed methodology and measures would work in the same way, with the value chain still structured around the smallest possible unit—in this case the enterprise. Despite the starting point of analysis of value chain structure being the smallest units of analysis, the approach offers many different aggregation possibilities to capture the changing economic structure of production as a whole.

14 The vertical and horizontal fragmentation of production is often represented with metaphors of snakes (sequential value transfers from one firm to further stages in a linear sequence) and spiders (simultaneous value transfers from different firms to the same company) (Baldwin and Venables 2013).

15 Technically, that would require I–O matrices of a dimension as large as the number of all firms of all countries included in such an international I–O structure.

16 Formal addition of further *n* dimensions to the usual $n \times n$ dimensions.

17 Definitions of all notations are given in Appendix  A.

18 The simplification consists only of the notation. We retain all the complexity of the block-matrix structure of the international I–O data and remove only the large number of indices, which would make the equations much more difficult to read.

19 Our downstream output decomposition formally coincides with the output decomposition of the approach that integrates output decomposition with a demand-driven decomposition of exports (Arto et al. 2019).

20 $CS_k$ represents an index for different country-sectors. $a_{ CS _1CS_2}$ thus represents a single Leontief technical coefficient indicating that the value produced by $CS_2$ requires a $a_{ CS _1CS_2}$ share of $CS_1$ input.

21 For example, Wang’s disaggregation into simple and complex GVCs uses the number of cross-border transactions, regardless of whether the value crossed a border for production or whether it is only an export to end users. Such a criterion mixes two conceptually different transactions, leading to unnecessary calculation complexity and the impossibility of further conceptual disaggregation. Existing definitions of the typology of value chains, like all such definitions, are constructed in a relatively arbitrary way. More important than strict adherence to the prevailing definitions is the clarity of the proposed revision and the presentation of the conceptual relationship of the new concepts with the old ones. Our proposal facilitates a more detailed decomposition that will allow researchers to construct an indicator better suited to their research questions. Since the revised typology is based on a more detailed decomposition compared to the currently prevailing typology, researchers can (by simply adding components of the revised decomposition) also replicate objects that correspond to existing studies.

22 Here we examine the path of production fragmentation, while the path to final consumption, which represents an additional transaction, is analysed in Sect. 3.2.5.

23 Details of the disaggregation are given in Appendix  B.

24 This is determined by the pure diagonal elements of the Leontief technical matrix *A*. Each $a_{ii}$ represents the portion of the total product of the *i*th country-sector that requires the use of the intermediate product of the same country-sector in the production process, thereby covering cyclical transactions within a sector. These cyclical transactions are of course included in the decomposition of the domestic value chain and not the no value chain since cyclical transactions represent the fragmentation of a domestic value chain.

25 The Greek and Italian economies, which experienced the longest recession in the EU during the period, experienced this very pattern (general reduction of production fragmentation, chiefly a reduction of domestic production fragmentation and increased integration into global value chains).

26 A variety of institutional and structural economic positions brings a range of effects of global integration on the country level.
